# Nicotinamide promotes cardiomyocyte derivation and survival through kinase inhibition in human pluripotent stem cells

**DOI:** 10.1038/s41419-021-04395-z

**Published:** 2021-11-29

**Authors:** Ya Meng, Chengcheng Song, Zhili Ren, Xiaohong Li, Xiangyu Yang, Nana Ai, Yang Yang, Dongjin Wang, Meixiao Zhan, Jiaxian Wang, Chon Lok LEI, Weiwei Liu, Wei Ge, Ligong Lu, Guokai Chen

**Affiliations:** 1 Zhuhai Precision Medical Center, Zhuhai People’s Hospital (Zhuhai hospital affiliated with Jinan University), Zhuhai, 519000 China; 2 Faculty of Health Sciences, University of Macau, Taipa, Macau, SAR China; 3 Centre of Reproduction, Development and Aging, Faculty of Health Sciences, University of Macau, Taipa, Macau, SAR China; 4 Guangdong Cardiovascular Institute, Guangdong Provincial Key Laboratory of South China Structural Heart Disease, Guangdong Provincial People’s Hospital, Guangdong Academy of Medical Sciences, Guangzhou, 510000 China; 5 School of Pharmaceutical Sciences & the Fifth Affiliated Hospital, Guangzhou Medical University, Guangzhou, 511436 China; 6 Department of Cardio-Thoracic Surgery, Nanjing Drum Tower Hospital, The Affiliated Hospital of Nanjing University Medical School, Nanjing, 210000 China; 7 HELP Stem Cell Innovations Ltd. Co, Nanjing, 210000 China; 8 Bioimaging and Stem Cell Core Facility, Faculty of Health Sciences, University of Macau, Taipa, Macau, SAR China; 9 MoE Frontiers Science Center for Precision Oncology, University of Macau, Taipa, Macau, SAR China

**Keywords:** Regenerative medicine, Kinases, Target identification, Stem-cell differentiation

## Abstract

Nicotinamide, the amide form of Vitamin B3, is a common nutrient supplement that plays important role in human fetal development. Nicotinamide has been widely used in clinical treatments, including the treatment of diseases during pregnancy. However, its impacts during embryogenesis have not been fully understood. In this study, we show that nicotinamide plays multiplex roles in mesoderm differentiation of human embryonic stem cells (hESCs). Nicotinamide promotes cardiomyocyte fate from mesoderm progenitor cells, and suppresses the emergence of other cell types. Independent of its functions in PARP and Sirtuin pathways, nicotinamide modulates differentiation through kinase inhibition. A KINOMEscan assay identifies 14 novel nicotinamide targets among 468 kinase candidates. We demonstrate that nicotinamide promotes cardiomyocyte differentiation through p38 MAP kinase inhibition. Furthermore, we show that nicotinamide enhances cardiomyocyte survival as a Rho-associated protein kinase (ROCK) inhibitor. This study reveals nicotinamide as a pleiotropic molecule that promotes the derivation and survival of cardiomyocytes, and it could become a useful tool for cardiomyocyte production for regenerative medicine. It also provides a theoretical foundation for physicians when nicotinamide is considered for treatments for pregnant women.

## Introduction

Vitamins are indispensable nutrients for daily functions in the human body. Vitamins are also essential for embryonic development, so they are often supplemented to pregnant women to reduce the risk of birth defects [1–4]. For example, Vitamin B3 supplement is prescribed to avert birth defects of spine and heart [5], indicating its potential roles in mesodermal differentiation. However, little is known about how vitamin B3 supplement could impact embryogenesis during the pregnancy.

Vitamin B3 is the precursor for ß-nicotinamide adenine dinucleotide (NAD^+^), which is essential in many oxidation and reduction reactions [6]. Vitamin B3 contains three family members, including niacin, nicotinamide, and nicotinamide riboside that possesses various additional functions regulating cellular physiology. Nicotinamide has been used at high doses to treat various diseases, such as schizophrenia, depression, Alzheimer’s disease, diabetes, and cancer [7–12]. Nicotinamide demonstrates beneficial effects in fetal development as insufficient nicotinamide intake increases the risk of congenital heart defects (CHD) at birth [13, 14]. At the same time, nicotinamide is often used to treat pregnant women with preeclampsia, a pregnancy complication characterized by high blood pressure [15]. Nicotinamide decreases blood pressure and endotheliosis in the mothers, and corrects the growth restriction in the pups [16]. Moreover, nicotinamide is implicated in the early development of mouse embryo in vitro [17]. Nicotinamide was often regarded as an inhibitor of PARP and Sirtuin pathways [12, 18]; however, there is limited evidence to directly support this role [11, 19–21]. We recently reveal that nicotinamide promotes stem cell survival and differentiation by inhibiting ROCK and CSNK1 kinases [22, 23]. Although it has been empirically utilized during pregnancy, it is essential to understand how nicotinamide regulates human embryonic development and stem cell differentiation to support its use in translational and clinical applications.

Human embryonic stem cells (hESCs) resemble post-implantation epiblast stem cells, and have the potential to differentiate to all cell types in the human body. Nicotinamide is beneficial for hESC survival after dissociation, and it also facilitates the differentiation into neural and pancreatic cell types [24–26]. Considering nicotinamide’s applications in preventing cardiovascular defects during pregnancy, we examine the molecular mechanism of nicotinamide in regulating mesodermal differentiation from hESCs in chemically defined conditions. Notably, nicotinamide alone is sufficient to induce cardiomyocyte differentiation, and it acts independently of the canonical WNT pathway. To understand the mechanism of nicotinamide, we identify 14 novel nicotinamide targets via a 468-kinase inhibition screen. Nicotinamide promotes cardiac differentiation through p38 MAP kinase inhibition. Besides that, nicotinamide also improves the survival of hESC-derived cardiomyocytes through ROCK inhibition. Our findings demonstrate that nicotinamide is a multi-functional modulator in cardiac differentiation and production, implying its potential impact on embryogenesis when it is prescribed as a nutrition supplement for pregnant women.

## Materials and methods

The experiments in this study were approved by the Institutional Review Board at University of Macau and Zhuhai People’s Hospital. All the experiments were performed following all the relevant and applicable governmental and institutional regulations and guidelines.

### Materials

H1 and H9 hESC lines were obtained from WiCell Institute, and human-induced pluripotent stem cell (hiPSC) NL4 was obtained from the National Institutes of Health, USA. H1 hESCs were used for most experiments unless otherwise stated. The usage of hESCs and hiPSCs was approved by the Institutional Review Board at the University of Macau. The materials used in this study were summarized in Supplementary Table 1.

### hPSC culture and maintenance

hPSCs were maintained in home-made E8 medium following the published protocol [27]. Briefly, hPSCs were cultured in E8 medium on matrigel-coated plates for 3−4 days, and then passaged using DPBS-EDTA. ROCK inhibitor Y-27632 (Selleck) 3 μM was applied to improve the cell viability during passaging.

### Mesoderm differentiation

hESCs were seeded on matrigel-coated plate and cultured for 2 days in E8 medium. All the experiments of differentiation were performed in the E5 basal medium which containing DMEM/F12 (Thermo), holo-transferrin (Sigma, 10 μg/mL), L-ascorbic acid-2-phosphate magnesium (Sigma, 64 μg/mL), sodium selenite (Sigma, 14 ng/mL) and chemically defined lipid concentrate (Gibco). When the cell confluency was about 60−80%, 5 μM CHIR99021 (Selleck) was applied for 1 day to induce mesoderm differentiation in E5 basal medium. In order to suppress spontaneous cardiac differentiation, 1 μg/mL insulin (Sigma) was added to E5 basal medium on day 1 for one day. Different treatments or combinations were added to the cells from day 2 to day 5 in E5 medium, including IWP-2 (Selleck, 3 μM), VEGF (Peprotech, 50 ng/mL), and nicotinamide (Sigma, 10−20 mM). Nicotinamide stock solution (1 M) was prepared under sterile conditions with DMEM/F12, and applied 1:100 (final 10 mM) or 1:50 (final 20 mM) to E5 medium. E5 medium with no treatment was used as control. After day 5, differentiated cells were cultured in E5 medium till day 7, and then 10 μg/mL insulin was added to the culture medium to maintain differentiated cells.

### Cardiac differentiation

After mesodermal induction by CHIR99021 on day 1, 3 μM IWP-2 was applied between day 2 and day 5 as positive control. Nicotinamide was added at the indicated times or doses to optimize the differentiation protocol. In routine practices, nicotinamide was added from day 1 to day 5. Then the modulators related to nicotinamide were used in cardiac differentiation from day 1 to day 5 unless otherwise mentioned. The concentration of modulators used in this study: Niacin (Sigma) 10 mM, EX527 (SIRT1i, Selleck) 10 μM, AZD2281 (PARPi, Selleck) 100 nM, Y27632 (ROCKi, Selleck) 10 μM, SB202190 (P38i, Selleck) 10 μM, D4476 (CSNK1i, Selleck) 10 μM or BIX02189 (MEK5i, Selleck) 5 μM.

### Zebrafish

The maintenance and larvae treatment of Tg (Fli1:EGFP) transgenic zebrafish were performed as previously reported [28]. The usage of transgenic zebrafish was approved by the Institutional Review Board at University of Macau. The transgenic zebrafish embryos were randomly subjected to 10 mM nicotinamide at 18−24 h post-fertilization. After 3 days of treatment, images of zebrafish were taken with SMZ18 (Nikon) Stereomicroscope.

### RNA extraction and qPCR

Total RNAs of hESCs and differentiated cells were isolated using RNAiso Plus (Takara), and then cDNA was synthesized with high-capacity cDNA reverse transcription kit (Thermo). qPCR was performed using SYBR Premix Ex Taq^TM^ (Takara) on QuantStudio^TM^ 7 Flex Real-Time PCR System. Target gene levels were normalized to TATA-Box Binding Protein (TBP) using delta−delta Ct method. qPCR primers were ordered from IDT, and primer sequences were listed in Supplementary Table 1.

### Flow cytometry

The cells were dissociated with TrypLE (Gibco) at the indicated time. After neutralization with 5% FBS, cells were fixed by 1% paraformaldehyde (Sigma) for 10 min at room temperature, and then permeabilized by 0.1% triton X-100 (Sigma) for 10 min. After washing, cells were incubated in the primary antibodies at 1:100 dilution for TNNT2 (DSHB) at 4 °C overnight. The cells were washed with PBS for three times before the incubation with specific secondary antibodies (goat-anti-mouse Alexa 488) at 1:500 dilution at room temperature for 30 min. Cells were then washed and resuspended in PBS for flow cytometry analyses by BD C6.

### Immunocytochemistry

Differentiated cells were washed with PBS and fixed with 4% paraformaldehyde for 10 min at room temperature, and then permeabilized by 0.1% triton X-100 (Sigma) for 10 min. After washing and blocking, cells were incubated in the primary antibodies of NKX2.5 (Santa cruz, sc-14033), TNNT2 (DSHB, CT3), α-Actinin (Abcam, ab68167) or β-Catenin (Santa cruz, sc-59737) at 1:100 dilution in 1% BSA at 4 °C overnight. Cells were washed with PBS for three times, and then incubated with alexa 488 conjugated goat anti-mouse antibody (Jackson, 115-545-071) and/or alexa 594 conjugated donkey anti-rabbit antibody (Jackson, 711-585-152) at 1:500 dilution for 1 h at room temperature. After removing the secondary antibodies, the cells were incubated with DAPI for 10 min at room temperature. Finally, the samples were washed three times and then mounted with vectashield (Vector Laboratories). Images were taken using Carl Zeiss Confocal LSM710 or Leica confocal SP8. Sarcomere length was analyzed using Leica Application Suite X (LAS X).

### Electrophysiology analysis

The electrophysiology analysis was performed by HELP Stem Cell Innovations Ltd. Company. Firstly, cardiomyocytes were plated on matrigel-coated slides in 24-well plate, and cultured for 2 days. Then current signals were recorded using EPC 10 USB—Heka Patch Clamp Amplifiers. Patch pipettes were prepared in a Sutter P-97 micropipette puller, and filled with internal fluid with the resistances of between 2 and 5 MΩ. The liquid junction potential was 5.946 mV, and the correction was performed by the EPC10 USB Amplifier (HEKA) automatically. All experiments were performed at 37 °C. The recordings were measured without external pacing, both types of cardiomyocytes exhibit spontaneous beating. PatchMaster software was used for data acquisition, and Clampfit software was applied for data analysis. The raw data was presented without further filtering, with a sampling rate of 20 kHz.

### Western blot

Cardiomyocytes were dissociated by TrypLE around day 10, and different doses of nicotinamide or ROCK inhibitor Y27632 10 μM were added in the medium for 1 h. The protein was harvested after 1 h for western blot [22]. Briefly, 40 μg protein of each sample was separated by electrophoresis with SDS-PAGE gels, and then transferred to PVDF membranes. The membranes were first blocked with 5% non-fat milk, and then incubated with antibodies against p-MLC (Ser19) 1:500 (Cell Signaling, 3671), MLC 1:1000 (Sigma, M4401), or Actin 1:2000 (Santa Cruz, sc-47778) overnight at 4 °C. After washing, the membranes were incubated with HRP-secondary antibodies (Jackson, 115-035-146 or 111-035-144) for 2 h at room temperature. Chemiluminescence was detected using SuperSignal^TM^ West Pico PLUS Chemiluminescent Substrate (Thermo) or West Dura Extended Duration Substrate (Thermo).

### Kinase screening

The kinase screening was performed by DiscoverX [22]. Briefly, all the kinases were expressed in HEK-293 cells or BL21 strain, and then DNA tags were added on the kinases to detect the binding affinity. Affinity beads were prepared by incubation of the streptavidin-coated magnetic beads with biotinylated ligands. After blocking and washing, the affinity resins were removed, and affinity beads with ligands were used in the binding reaction together with test compounds and kinases. After reactions, the beads were washed and eluted, and kinase concentration was determined by qPCR. Binding activities of nicotinamide with MEK5, P38α, and P38γ were determined, and 9 doses of nicotinamide with three-fold dilution were used with the same method.

% Ctrl calculation

$$[\frac{{{{{\mathrm{test}}}}\;{{{\mathrm{compound}}}}\;{{{\mathrm{signal}}}} - {{{\mathrm{positive}}}}\;{{{\mathrm{control}}}}\;{{{\mathrm{signal}}}}}}{{{{{\mathrm{negative}}}}\;{{{\mathrm{control}}}}\;{{{\mathrm{signal}}}} - {{{\mathrm{positive}}}}\;{{{\mathrm{control}}}}\;{{{\mathrm{signal}}}}}}] \times 100$$

Test compound: nicotinamide

Negative control: DMSO (100% Ctrl)

Positive control: The specific kinase inhibitors were used as control compound (0% Ctrl)

### P38δ activity measurement

The measurement was performed following the protocol provided by Promega. Briefly, 50 ng P38δ, 5 μM ATP, 0.5 μg substrate, and inhibitors were added for the reaction in 96-well plate. The final concentration of SB202190 was 10 μM, and 20 mM nicotinamide was diluted by three fold for 8 points in the reaction. After 1 h incubation at room temperature, 25 μL ADP-Glo^TM^ reagent was added, and reacted for 40 min at room temperature. Then 50 μL kinase detection reagent was added, followed by 30 min of incubation at room temperature. Luminescence was examined using PerkinElmer Victor X3 Microplate Reader.

% P38δ activity calculation

$$[\frac{{{{{\mathrm{test}}}}\;{{{\mathrm{compound}}}}\;{{{\mathrm{signal}}}} - {{{\mathrm{background}}}}\;{{{\mathrm{signal}}}}}}{{{{{\mathrm{negative}}}}\;{{{\mathrm{control}}}}\;{{{\mathrm{signal}}}} - {{{\mathrm{background}}}}\;{{{\mathrm{signal}}}}}}] \times 100$$

Test compound signal: reaction with nicotinamide or SB202190

Background signal: reaction without substrate

Negative control signal: reaction without inhibitor.

### Microarray

The microarray experiment was performed following the previous publication [29]. H1 cells were treated with or without nicotinamide for 3 days, and RNA was isolated using RNAiso Plus. Then cRNA was synthetized with SuperScript III kit and TargetAmp™-Nano Labeling Kit (Epibio). Samples were hybridized using HumanHT-12 v4 Expression BeadChip Kit (Illumina), and then the RNA chip slides were examined by the iScanner (Illumina). The differentiated cells induced by IWP-2, nicotinamide, or SB202190 were harvested after 10 days of differentiation, and RNA was extracted and performed for microarray with the same method.

### Bioinformatics analyses

The data of microarray were analyzed based on the method reported previously [29]. Briefly, data were processed using the arrayanalysis.org portal (www.arrayanalysis.org). Genes with a fold change ≥ 1.5 or 2 were considered differentially expressed. Heatmap was generated using the {pheatmap} package in R. Gene ontology and KEGG pathway enrichment analyses were performed through Database for Annotation, Visualization and Integrated Discovery (DAVID) (https://davidd.ncifcrf.gov/) version 6.7. The cell type analysis was performed by Enrichr (http://amp.pharm.mssm.edu/Enrichr/). The accession numbers for the microarray data in this study is GEO: GSE154455.

### Statistical analysis

The heatmap with clustering was created by R 3.4.2 software, and other heatmaps were generated by GraphPad Prism 7. Data are shown as mean ± SD of three independent experiments unless otherwise specified. Two-tailed Student’s t-test was performed for statistical analysis, and *p* < 0.05 was considered statistically significant.

## Results

### Nicotinamide promotes cardiomyocyte differentiation from mesoderm progenitor cells

In order to understand the molecular mechanism of nicotinamide in human embryonic development, we analyzed global gene expression data of hESCs treated with nicotinamide in E8 maintenance medium in our previous report [22]. Nicotinamide modulated the gene expression that is involved in the development of various organs such as heart, vascular system, brain, and kidney (Supplementary Fig. 1A, B). These data suggest that nicotinamide could play active roles in hESC fate determination in multiple lineages.

**Fig. 1:**
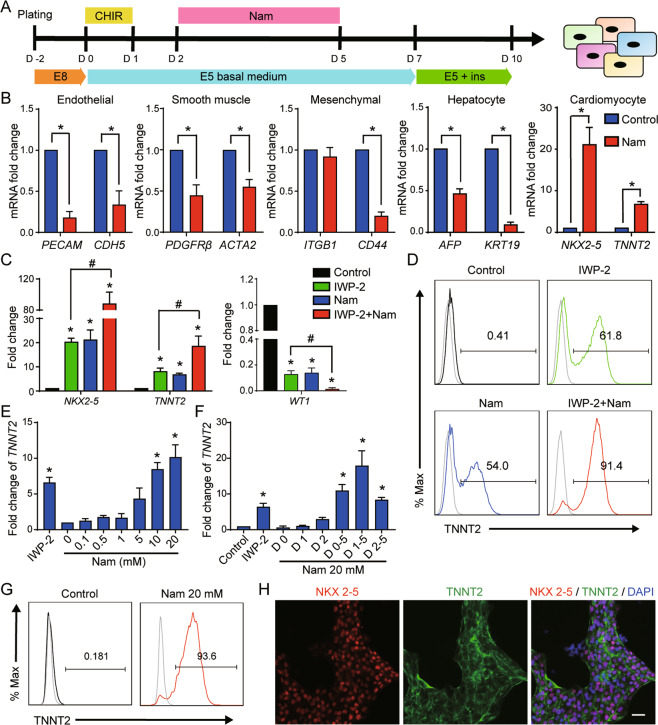
Effect of nicotinamide on mesodermal differentiation. **A** The schematic diagram showing the protocol of mesoderm differentiation with or without nicotinamide. hESCs were seeded in matrigel-coated plates for 2 days, treated with CHIR99021 (CHIR) 5 μM for 1 day, and then treated with or without nicotinamide 10 mM from day 2 to day 5. Differentiated cells were harvested for analyses at day 10. **B** Analyses of mRNA expression levels of *PECAM*, *CDH5*, *PDGFRB*, *ACTA2*, *ITGB1*, *CD44*, *AFP*, *KRT19*, *NKX2-5*, and *TNNT2* in the neutral condition with or without nicotinamide treatment. Blue, control; red, nicotinamide 10 mM. **C** Analyses of mRNA expression levels of *NKX2-5*, *TNNT2*, and *WT1* in cardiac differentiation. Black, Control; green, IWP-2 3 μM; blue, Nicotinamide 10 mM; red, IWP-2 3 μM and nicotinamide 10 mM. **D** The percentage of TNNT2-positive cells was determined by flow cytometry. The results shown are representative of three independent experiments. Gray line indicates the level of isotype control. **E** Dose-dependent effect of nicotinamide on the gene expression of *TNNT2* in cardiac differentiation. Nicotinamide or IWP-2 was added from day 2 to day 5. Data shown are normalized with Nam 0 (*p < 0.05 compared with Nam 0). **F** Timing effect of nicotinamide on the gene expression of *TNNT2* in cardiac differentiation. **G** After 13 days of differentiation, TNNT2-positive cells were determined by flow cytometry. Nicotinamide (Nam) at 20 mM added from day 1 to day 5. Gray line indicates the level of isotype control. The results shown are representative of 3 independent experiments. **H** Confocal microscopy images showing immunostaining of NKX2.5 and TNNT2 in differentiated cells treated by nicotinamide (Nam) 20 mM at day 13 of differentiation. Scale bar, 20 μm. Data shown are mean ± SD of three independent experiments (*p < 0.05 compared with control, #*p* < 0.05 compared with IWP-2).

It is unclear how nicotinamide influences mesoderm differentiation, so we inspected its impact on mesodermal progenitor cells. Mesoderm progenitor cells were induced from hESCs through WNT pathway activation by GSK3β inhibitor CHIR99021, and they could then further differentiate to somatic cell types including endothelial cells, smooth muscle cells, mesenchymal stem cells, hepatocytes, and cardiomyocytes (Fig. 1A, B). When nicotinamide was added from day 2 to day 5, cardiomyocyte gene expression was elevated, but other cell types were suppressed (Fig. 1B). Then we compared the impact of nicotinamide with common cardiac inducer IWP-2. Nicotinamide or IWP-2 alone was sufficient to induce cardiomyocyte differentiation with the similar expression level of cardiomyocyte markers *TNNT2* and *NKX2-5* (Fig. 1C, D). When the two compounds were applied together, they synergistically promoted cardiomyocyte differentiation, and suppressed epicardium marker gene *WT1* expression (Fig. 1C). This synergistic effect was also observed in hESC H9 and iPSC NL4 cell lines. (Supplementary Fig. 1C, D). These results suggest that nicotinamide might induce cardiomyocyte differentiation through a distinctive pathway from WNT inhibition, and the nicotinamide-associated pathway can complement WNT inhibition to improve cardiac differentiation in defined conditions.

To understand the dynamics of nicotinamide regulation in cardiac differentiation, we examined the effects of nicotinamide dose and treatment timing. Nicotinamide induced cardiomyocyte differentiation in a dose-dependent manner (Fig. 1E). We also found that nicotinamide was effective within a specific time frame. The strongest gene expression of *TNNT2* was achieved when nicotinamide was applied from day 1 to day 5 (Fig. 1F). Therefore, we added nicotinamide at 20 mM from day 1 to day 5 of differentiation in this study if not specified. Under nicotinamide treatment, beating cardiomyocytes were usually observed on day 7 or day 8 (Supplementary Video). Flow cytometry and immunostaining analysis showed that high-purity cardiomyocytes were induced by nicotinamide treatment (Fig. 1G, H). The method for cardiomyocyte induction was also effective in H9 hESCs and NL4 hiPSCs (Supplementary Fig. 1E, F). These data showed that nicotinamide could serve as an effective inducer in general cardiomyocyte production.

### Functional analysis of nicotinamide-derived cardiomyocytes

**Fig. 2:**
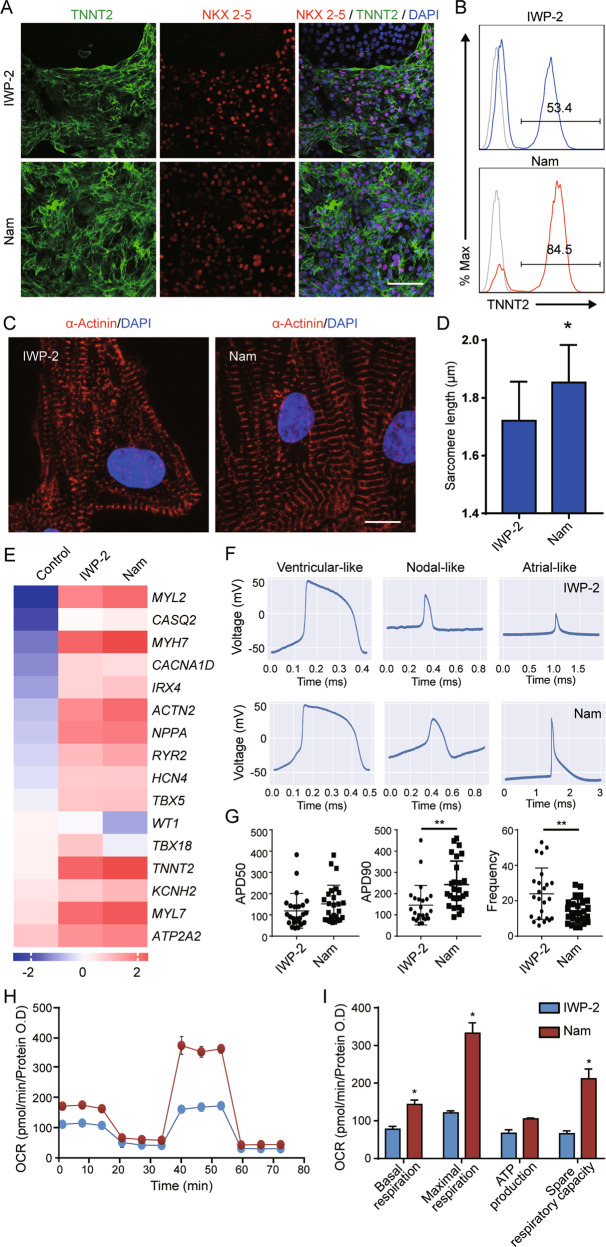
Characterization of cardiomyocytes induced by nicotinamide. **A** Immunostaining images of NKX2.5 and TNNT2 in differentiated cells treated by IWP-2 or nicotinamide (Nam) at day 30 of differentiation. Scale bar, 50 μm. **B** Representative flow cytometric analyses showing the proportion of TNNT2 at day 30 of differentiation. Gray line indicates the level of isotype control. Data are representative of three independent experiments. **C** Representative confocal microscopy images of α-Actinin and DAPI in the cardiomyocytes derived by IWP-2 3 μM or nicotinamide 20 mM after 25 days of differentiation. Scale bar, 10 μm. **D** Quantification of sarcomere length using LAS X software. (Data shown are mean ± SD, *n* = 38−40 cells, *p < 0.05 compared with IWP-2 group). **E** Heatmap showing the gene expression profiles of cardiomyocytes induced by IWP-2 or nicotinamide (Nam) at day 25. The differentiated cells were harvested at day 25, and the relative expression level of genes was analyzed by qPCR. Results shown are average of three independent experiments, and represent log10 of gene expression levels relative to TBP. **F** Representative recordings of active potential in nicotinamide or IWP-2-derived cardiomyocytes. **G** Action potential (AP) duration 50%, 90% (APD50, APD90), and frequency were analyzed. (Data shown are mean ± SD, *n* = 24−26 cells, *p < 0.05 compared with IWP-2 group). **H** Oxygen consumption rate (OCR) of IWP-2 or nicotinamide-induced cardiomyocytes was measured using Mito Stress Test. **I** Basal respiration, maximal respiration, ATP production, and spare respiratory capacity of cardiomyocytes were calculated. (Data shown are mean ± SD, *n* = 3−4, *p < 0.05 compared with IWP-2 group).

To analyze the biological functions of cardiomyocytes induced by nicotinamide, we maintained those cells for more than 25 days. After the extended culture, most cells remained positive for NKX2-5 and TNNT2 expression (Fig. 2A, B). We showed that α-Actinin-positive sarcomere structures emerged in cardiomyocytes generated from nicotinamide and IWP-2 methods (Fig. 2C). Longer sarcomeres were observed in nicotinamide-induced cardiomyocytes than IWP-2-induced cells (Fig. 2D). qPCR analysis showed that maturation markers were expressed in both types of cardiomyocytes (Fig. 2E), including *MYL7*, *MYL2*, *MYH7*, and *ATP2A2*, and ion channels *RYR2*, *CASQ2*, and *KCNH2* [30]. Meanwhile, expressions of epicardium markers *TBX18* and *WT1* were suppressed in nicotinamide-treated cells (Fig. 2E). Patch-clamp analysis showed that nicotinamide-induced cardiomyocytes had typical atrial, ventricular and nodal electrophysiology profiles (Fig. 2F). The electrophysiology study showed that nicotinamide-treated cardiomyocytes had longer APD90 and lower frequency than IWP-2-derived cardiomyocytes (Fig. 2G). Analysis using multi-electrode array (MEA) showed that cardiomyocyte induced by nicotinamide had higher conduction velocity and lower maximum propagation delay than IWP-2 induced cardiomyocytes (Supplementary Fig. 2). To analyze the mitochondrial function of cardiomyocytes, we performed mito stress test using Seahorse extracellular flux analyzer. Nicotinamide-treated cardiomyocytes had higher basal and maximal respiration and spare respiratory capacity than the IWP-2 group (Fig. 2H, I). These data demonstrated that functional cardiomyocytes were induced by nicotinamide.

### Nicotinamide promotes cardiac differentiation through P38 inhibition

**Fig. 3:**
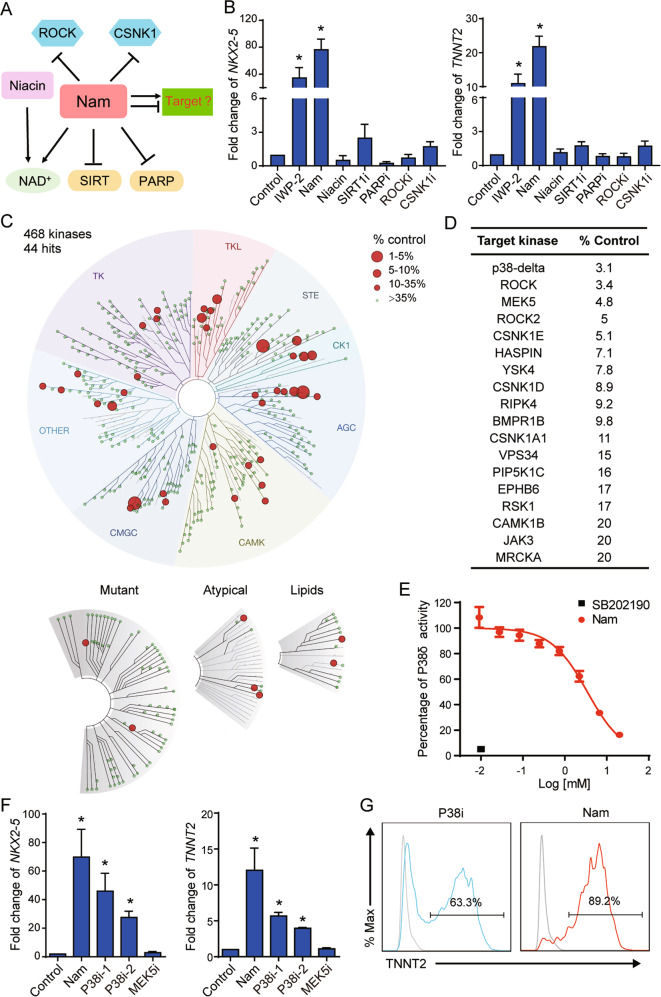
Nicotinamide directs cardiomyocyte cell fate through the inhibition of P38δ. **A** The diagram showing the molecules related to nicotinamide. Nicotinamide (Nam) and niacin can be transformed to NAD^+^ by different enzymes. Nicotinamide blocks the activation of these NAD^+^ consuming enzymes such as SIRT and PARP, and it also inhibits the activity of ROCK and CSNK1. **B** Analyses of mRNA expression levels of *NKX2-5* and *TNNT2* at day 10 of differentiation with the indicated treatments. hESC H1 cells were treated with IWP-2 3 μM from day 2 to day 5, Nicotinamide (Nam) 20 mM, Niacin 10 mM, EX527 (SIRT1i) 10 μM, AZD2281 (PARPi) 100 nM, Y-27632 (ROCKi) 10 μM or D4476 (CSNK1i) 10 μM from day 1 to day 5. **C** TREEspot^TM^ interaction maps of nicotinamide. % Control indicates the binding efficiency between the substrate and specific kinase, and lower numbers indicate stronger inhibition. Kinase hits (% Control < 35) are labeled with red circles, and larger circles indicate the kinases with lower % Control. **D** The target kinases with % Control < 20 under 3 mM nicotinamide treatment from the DiscoverX KINOMEscan service. Lower numbers of % Control indicates higher-affinity binding. **E** The influence of nicotinamide on P38δ activity in vitro measured by the bioluminescent kinase assay (*n* = 3 technical replicates). The red circle indicates different doses of nicotinamide (Nam), and the black square indicates SB202190 (P38i) 10 μM. The data shown are representative of three independent experiments. **F** Analyses of mRNA expression levels of *NKX2-5* and *TNNT2* on day 10 of differentiation treated with Nicotinamide (Nam) 20 mM, SB202190 (P38i-1) 10 μM, SB203580 (P38i-2) 10 μM, or BIX02189 (MEK5i) 5 μM from day 1 to day 5. **G** Representative flow cytometric analyses showing the percentage of TNNT2-positive cells after 13 days of differentiation induced by SB202190 (P38i) or nicotinamide (Nam). Results are representative of three independent experiments. Data shown are normalized to control, and presented as mean ± SD of three independent experiments (*p < 0.05 compared with Control).

In order to understand the molecular mechanism of the nicotinamide-associated cardiac induction, we examined which nicotinamide related pathways were involved in cardiac differentiation (Fig. 3A), but none of the pathway modulators enhanced cardiac differentiation including niacin, SIRT1 inhibitor, PARP inhibitor, ROCK inhibitor, and CSNK1 inhibitor (Fig. 3B). Nicotinamide is a known HDAC inhibitor, so we tested the influence of other HDAC inhibitor in differentiation, and found that HDAC inhibitor VPA could not induce cardiomyocytes, either (Supplementary Fig. 3A, B). These results indicated that novel targets were modulated by nicotinamide in cardiomyocyte differentiation.

We hypothesized that additional kinases could be targeted by nicotinamide to regulate stem cell differentiation, so we expanded the kinase inhibition screening to 468 kinases (Fig. 3C and Supplementary Table 2). 44 kinases out of 468 total targets had their activity inhibited for more than 65% by nicotinamide, and more than 80% of activities were suppressed in 18 kinases (Fig. 3C, D). Among the 18 kinases, ROCK1, ROCK2, CSNK1, and CSNK2 were previously reported [22], and the other 14 kinases were novel kinase targets by nicotinamide. Among these 14 kinases, P38δ and MEK5 were the most sensitive to nicotinamide inhibition (Fig. 3D). The bioluminescent kinase assay demonstrated that nicotinamide inhibited P38δ activity in vitro in a dose-dependent manner, while common P38 inhibitor SB202190 was sufficient to inhibit P38δ activity at 10 μM (Fig. 3E). These data imply that nicotinamide could act through those novel kinase targets to regulate cardiac differentiation.

In order to dissect nicotinamide function in mesoderm differentiation, P38 inhibitor SB202190, SB203580, and MEK5 inhibitor BIX02189 were added to mesoderm progenitor cells from day 1 to day 5. Cardiac markers *NKX2-5* and *TNNT2* were induced by P38 inhibitors SB202190 and SB203580, but not by MEK5 inhibitor (Fig. 3F). Flow cytometry confirmed that TNNT2-positive cardiomyocytes were generated under P38 inhibition (Fig. 3G). The shRNA lentivirus targeting *MAPK11* (*P38β*), *MAPK13* (*P38δ*), *MAPK14* (*P38α*) also improved cardiac induction (Supplementary Fig. 3C−I). These data indicate that nicotinamide promotes cardiac differentiation by inhibiting P38.

### Nicotinamide and P38 inhibition promote cardiac differentiation through β-catenin independent pathway

**Fig. 4:**
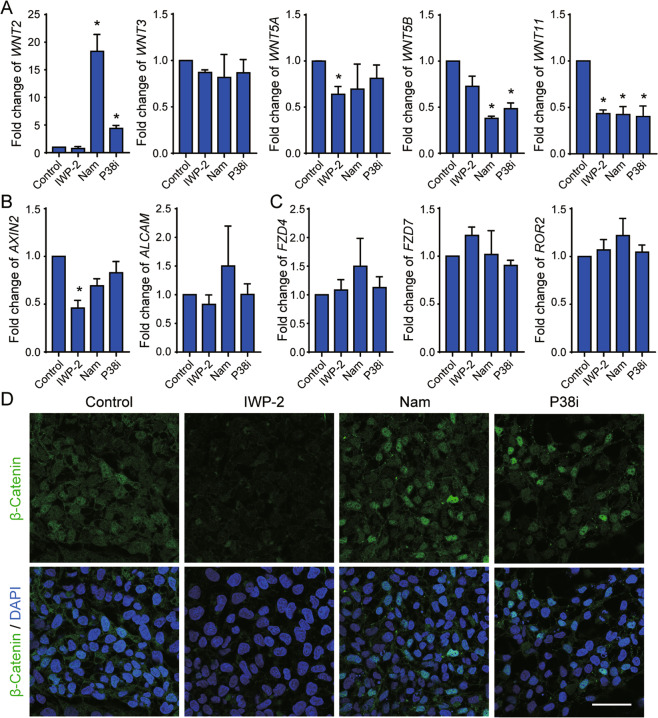
Nicotinamide-induced cardiac differentiation is independent of canonical WNT inhibition. **A** Analyses of gene expression of *WNTs* after 5 days of differentiation by qPCR. **B** Gene expressions of the downstream targets of canonical WNT (*AXIN2*) and noncanonical WNT (*ALCAM*) after 5 days of differentiation. **C** Gene expression of WNT receptors after 5 days of differentiation. **D** Confocal microscopy images showing the immunostaining images of β-Catenin in differentiated cells treated with IWP-2, nicotinamide (Nam), or P38 inhibitor (P38i). After plating for 2 days, hESCs were treated with CHIR99021 5 μM for 1 day, and then IWP-2 (3 μM), nicotinamide (20 mM) or P38 inhibitor SB202190 (10 μM) for 1 day to examine their impact on β-Catenin expression. Scale bar, 50 μm. Data shown are normalized to control, and represent mean ± SD of three independent experiments (*p < 0.05 compared with Control).

To test the impact of nicotinamide on WNT pathway, we first examined WNT gene expression, and found that they were differentially regulated under IWP-2, nicotinamide, and p38 inhibitor treatments after 5 days of differentiation (Fig. 4A). P38 inhibitor and nicotinamide specially elevated noncanonical *WNT2* expression, but suppressed expression of *WNT5B* (Fig. 4A). All three treatments suppressed *WNT11* expression 5 days after the differentiation induction (Fig. 4A). However, *AXIN2*, the canonically WNT downstream target, was repressed more significantly by IWP-2 than nicotinamide and p38 inhibitor, while another WNT/JNK pathway downstream gene *ALCAM* was not significantly influenced by the three treatments (Fig. 4B). We also found that the expression levels of WNT receptors did not show significant differences under the three treatments (Fig. 4C). Besides that, the accumulation of β-Catenin was suppressed by IWP-2, but not by nicotinamide or P38 inhibitor (Fig. 4D). These results support the notion that nicotinamide and p38 inhibition induced cardiac differentiation independent of canonical WNT inhibition.

### Comparison of global gene expressions in cardiomyocytes induced by nicotinamide, P38 inhibitor or IWP-2

**Fig. 5:**
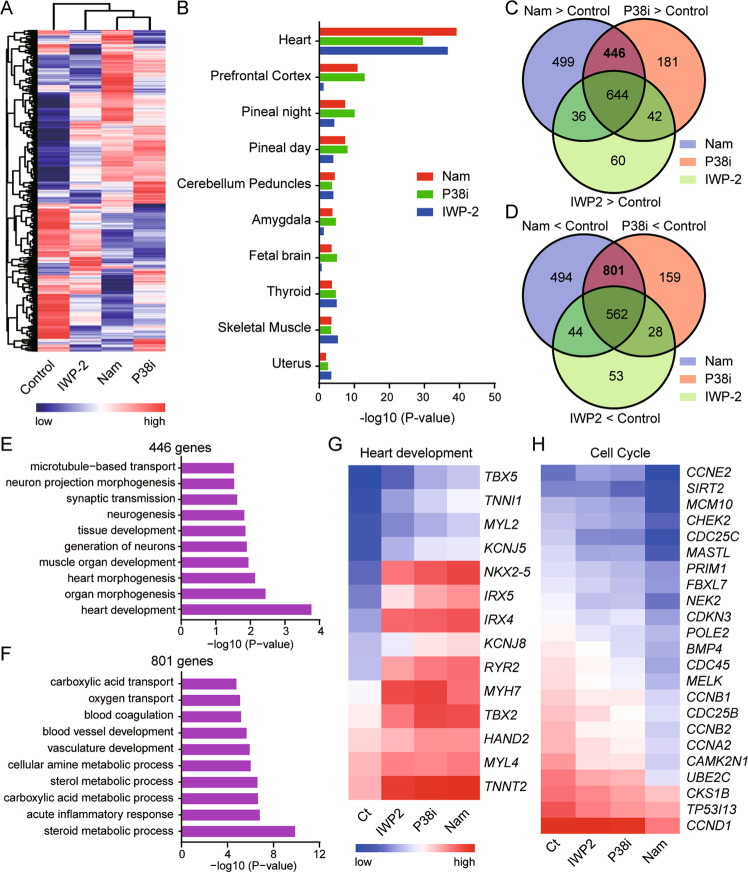
Comparison of gene expression in cardiomyocytes induced by WNT inhibitor, P38 inhibitor, and nicotinamide. **A** Hierarchical clustering analyses of differentiated cells with the indicated treatments. IWP-2 at 3 μM was added from day 2 to day5, and nicotinamide (Nam) at 20 mM or P38 inhibitor SB202190 (P38i) 10 μM were added from day 1 to day 5. The samples were harvested for microarray after 10 days of differentiation. **B** Enrichr analysis results showing the top 10 cell types enriched by nicotinamide, P38 inhibitor, or IWP-2 based on gene expression profiles. Venn diagram showing the numbers of genes upregulated (**C**) and downregulated (**D**) by nicotinamide (Nam), SB202190, or IWP-2 compared with untreated control. **E** The top 10 pathways enriched by the 446 genes upregulated by nicotinamide (Nam) and P38 inhibitor (P38i), but not IWP-2. **F** The top 10 pathways enriched by the 801 genes downregulated by nicotinamide (Nam), or P38 inhibitor (P38i), but not IWP-2. Comparison of gene expression related to heart development (**G**) and cell cycle (**H**) in the differentiated cells generated under different treatments after 10 days of differentiation.

To determine whether different induction methods lead to distinct molecular features in cardiomyocytes, we compared their global gene expression profiles by microarray. Hierarchy clustering analysis showed that IWP-2, nicotinamide, and P38 inhibitor led to more closely associated gene expressions compared to the neutral condition, while nicotinamide- and P38 inhibitor-treated cells clustered more closely (Fig. 5A). We then analyzed the upregulated genes in the group of IWP-2, nicotinamide, or P38 inhibitor by Enrichr [31], and the results showed that IWP-2, nicotinamide, or P38 inhibitor can all activate the expression of heart cell markers, but differentiation under nicotinamide or P38 inhibitor treatment generated more ectoderm cell types, such as prefrontal cortex, amygdala, and fetal brain, compared to IWP-2 (Fig. 5B). Furthermore, we analyzed the genes expressed differentially in nicotinamide, P38 inhibitor, and IWP-2 group by Venn diagram. Nicotinamide, P38 inhibitor, and IWP-2 increased the expression of 644 genes that were enriched in cardiac muscle contraction, morphogenesis, and development (Fig. 5C and Supplementary Fig. 4A). Meanwhile, the three treatments decreased the expression of 562 genes, which were enriched in blood coagulation, lipid homeostasis, vasculature development, and lipid metabolic process (Fig. 5D and Supplementary Fig. 4B). Venn diagram also shows nicotinamide and P38 inhibitor share more common differentially expressed genes than with IWP-2 (Fig. 5C, D). 446 genes were upregulated only by nicotinamide or P38 inhibitor but not by IWP-2, and KEGG analysis showed that these genes were involved in the pathways of heart development and heart morphogenesis (Fig. 5E). And the 801 genes specifically downregulated by nicotinamide or P38 inhibitor were related to the pathways of steroid metabolic process, blood vessel development, and inflammatory response (Fig. 5F). By examining the expression of genes related to heart development, we found that nicotinamide and P38 inhibitor induced higher level of *TBX5*, *TNNI1*, *MYL2*, *KCNJ5*, *KCNJ8*, *IRX5,* and *TBX2* than IWP-2 (Fig. 5G). This result supports our model that nicotinamide and P38 inhibitor induce cardiomyocytes through p38 inhibition.

However, nicotinamide itself increased 499 genes related to mitochondrial electron transport and cellular respiration (Supplementary Fig. 4C) and decreased 494 genes strongly enriched to cell cycle regulation (Supplementary Fig. 4D). Then we examine the genes related to mitochondrial electron transport, and nicotinamide specially upregulated *NDUFA13*, *ETFB*, *NDUFB7*, *NDUFA3*, *NDUFB9*, *ATP5J*, and *NDUFS3* (Supplementary Fig. 4E). And interestingly, the cardiomyocytes generated by nicotinamide treatment expressed lower levels of cell cycle-related genes compared with other groups (Fig. 5H). These results indicate that cardiomyocytes derived by nicotinamide possibly have high mitochondrial activity and low proliferation ability, and an additional nicotinamide target is involved in its regulation of cardiac differentiation.

### Nicotinamide improves the survival of individualized cardiomyocytes through ROCK inhibition

**Fig. 6:**
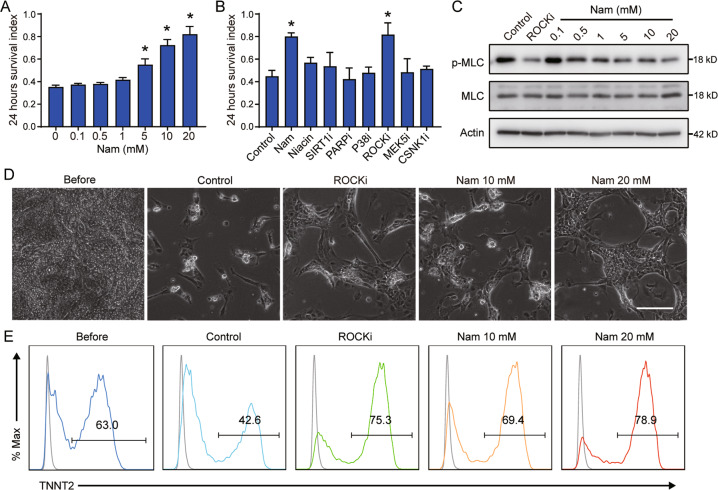
Nicotinamide promotes the survival of hESC-derived cardiomyocytes and improves cardiomyocyte purity. **A** Dose-dependent impact of nicotinamide on the survival of hESC-derived cardiomyocytes. After 10 days of differentiation, hESC-derived cardiomyocytes were passaged under the indicated doses of nicotinamide (Nam). The survival index indicates the number of living cells divided by the input cells after 24 h of plating. **B** The effect of different modulators related to nicotinamide on cardiomyocyte survival 24 h after dissociation. Nicotinamide (Nam) 20 mM, Niacin 20 mM, EX527 (SIRT1i) 10 μM, AZD2281 (PARPi) 100 nM, SB202190 (P38i) 10 μM, Y-27632 (ROCKi) 10 μM, BIX02189 (MEK5i) 5 μM or D4476 (CSNK1i) 10 μM. **C** Phosphorylation of MLC (Ser19) in dissociated cardiomyocytes under indicated doses of nicotinamide or ROCK inhibitor (Y27632, 10 μM) treatment for 1 h after individualization. **D** Representative phase-contrast images of cardiomyocytes passaged under the indicated treatments, 24 h after plating. Nicotinamide (Nam) 10 mM or 20 mM, Y27632 (ROCKi) 10 μM. Scale bar, 100 μm. **E** Flow cytometric analyses showing the percentage of TNNT2-positive cardiomyocytes passaged under the indicated treatments, 24 h after plating. Data shown are normalized to control, and represent mean ± SD of three independent experiments (*p < 0.05 compared with Control).

In order to utilize hESC-derived cardiomyocytes in regenerative medicine, these cells have to be effectively harvested and stored without severe cell death or the loss of cell purity. In the freshly derived cardiomyocytes, more than half cardiomyocytes died within 24 h after dissociation and passaging (Fig. 6A). The cell death phenotype was suppressed by nicotinamide in a dose-dependent manner (Fig. 6A). We inspected the potential targets involved in cardiomyocyte survival. After dissociation and passaging, only ROCK inhibitor Y27632 was able to rescue cell survival similar to nicotinamide, while other inhibitors of nicotinamide targets failed to improve cell survival (Fig. 6B). Myosin light chain (MLC) is the downstream target of ROCK, so we measured the phosphorylation of MLC after cardiomyocyte dissociation to confirm the inhibition of ROCK activity by nicotinamide. ROCK inhibitor or nicotinamide suppressed MLC phosphorylation after 1 h of cell individualization (Fig. 6C and Supplemental Fig. 5A). Inhibition of ROCK can lead to autophagy impairment or activation [32, 33], so we examined the influence of nicotinamide or ROCK inhibitor on the autophagy flux during cardiomyocyte dissociation. However, neither nicotinamide nor ROCK inhibitor had a significant effect on autophagy markers LC3 and P62 in hESC-derived cardiomyocytes (Supplemental Fig. 5B−E). These data suggest that nicotinamide improves cardiomyocytes survival through the inhibition of ROCK kinase, but independently of autophagy regulation. We further confirmed that nicotinamide was beneficial to cardiomyocyte survival but not to other cell type during passaging (Fig. 6D, E). The bright field images showed that nicotinamide and Y27632 promoted cardiomyocytes survival after 24 h of dissociation (Fig. 6D). Flow cytometry analysis showed that ROCK inhibitor or nicotinamide was sufficient to promote cell survival and maintain the TNNT2-positive ratio (Fig. 6E). Furthermore, nicotinamide and ROCK inhibitor also had a similar effect on the survival of cardiomyocytes induced by nicotinamide (Supplementary Fig. 5F, G).

### Nicotinamide affects heart development, but has no effect on vascular system formation during fish development

**Fig. 7:**
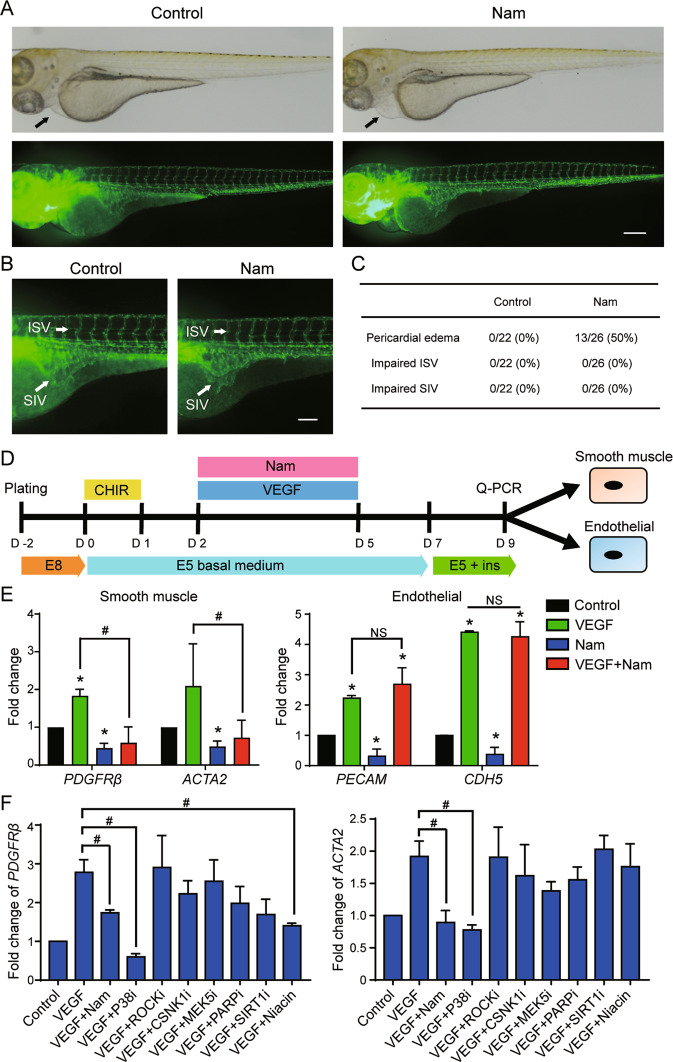
The influence of nicotinamide in zebrafish cardiovascular system development. **A** Effect of nicotinamide on the development of The Tg(Fli1:EGFP) transgenic zebrafish. Nicotinamide was applied between 18 and 24 h post fertilization. The images were taken after 3 days of treatment. Top, the bright field images of zebrafishes; Bottom, the fluorescence images. Scale bar, 200 μm. **B** The enlarged images showing the intersegmental vessels (ISV) and subintestinal veins (SIV) of zebrafishes. Scale bar, 100 μm. **C** Percentage of zebrafish with pericardial edema, impaired ISV, or impaired SIV (*n* = 22 in control group, and *n* = 26 in nicotinamide group). **D** The schematic diagram showing the protocol of endothelial and smooth muscle differentiation. VEGF 50 ng/mL was added from day 2 to day 5 in the absence or presence of 10 mM nicotinamide. **E** Analyses of mRNA expression levels of *PECAM*, *CDH5*, *PDGFRβ*, and *ACTA2* by qPCR at day 9 of differentiation. Black, Control; green, VEGF 50 ng/mL; blue, Nicotinamide 10 mM; red, VEGF 50 ng/mL and nicotinamide 10 mM. Data shown are normalized to control and represent three independent experiments (*p < 0.05 compared with control, #*p* < 0.05 compared with VEGF). **F** The indicated inhibitors were added to analyze the mechanism of nicotinamide in VEGF-induced differentiation. The mRNA levels of *PDGFRβ* and *ACTA2* were measured by q-PCR at day 9 of differentiation. The following treatments were added from day 2 to day 5 together with VEGF (50 ng/mL): Nicotinamide (Nam,10 mM), P38 inhibitor SB202190 (P38i, 10 μM), ROCK inhibitor Y-27632 (ROCKi, 10 μM), CSNK1 inhibitor D4476 (CSNK1i, 10 μM), MEK5 inhibitor BIX02189 (MEK5i, 5 μM), PARP inhibitor AZD2281 (PARPi, 100 nM), SIRT1 inhibitor EX527 (SIRT1i, 10 μM), or Niacin (10 mM). Data shown are normalized to control and represent mean ± SD of three independent experiments (#*p* < 0.05 compared with VEGF).

To study the influence of nicotinamide in mesodermal development in vivo, we examined the impact of nicotinamide on vascular development in a zebrafish model (Tg (Fli1:EGFP)) which expresses *fli1* driven EGFP in the vascular vessels [34]. Tg (Fli1:EGFP) zebrafish embryos were subjected to nicotinamide treatment for 3 days before microscopic examination. Half of the embryos exposed to nicotinamide displayed enlarged heart, but normal vascular systems were observed in all the animals (Fig. 7A−C). This result suggested that other factors could modulate in vivo endothelial differentiation in the presence of nicotinamide, which prevents nicotinamide from interfering with endothelial differentiation during embryogenesis. To test this hypothesis, endothelial inducing factor VEGF was applied along with nicotinamide to examine its impact on endothelial differentiation from hESCs (Fig. 7D). VEGF alone enhanced both endothelial and smooth muscle differentiation, while nicotinamide alone suppressed both cell types (Fig. 7E). To explore the mechanism of nicotinamide in VEGF-induced differentiation, we added the nicotinamide-related inhibitors together with VEGF. The expression of *PDGFRβ* and *ACTA2* was significantly decreased by nicotinamide or P38 inhibitor (Fig. 7F), but the endothelial markers *PECAM* and *CDH5* were not changed (Supplementary Fig. 6). These data suggest that nicotinamide is able to induce cardiomyocyte differentiation, and it can also crosstalk with the VEGF signaling pathway through P38 inhibition during embryogenesis.

## Discussion

Nicotinamide is not only a vitamin involved in metabolism, but also a therapeutic agent for various diseases. Nicotinamide is prescribed to reduce birth defects and treat preeclampsia in pregnant women [5, 16]. However, the molecular mechanisms of nicotinamide during embryogenesis have not been completely understood. This study reveals nicotinamide as a cardiomyocyte inducer through kinase inhibition, and provides efficient methods to derive and maintain cardiomyocytes from hESCs in chemically defined conditions.

In vertebrate heart development, WNT pathway plays pivotal roles in cardiomyocyte cell fate determination. The temporal modulation of WNT pathway has been widely used to induce cardiomyocytes from hESCs [35–37]. In most protocols, serum albumin is required to enhance cardiac differentiation based on WNT modulation, which limits the usage of hESC-derived cardiomyocytes in regenerative therapy [37–39]. We develop an efficient method with nicotinamide to generate functional human cardiomyocytes in albumin-free conditions, which is independent of canonical WNT pathway. Furthermore, our study provides a model to investigate the mechanism of human cardiomyocyte development beyond canonical WNT pathway.

Nicotinamide has been widely used in stem cell study, such as pancreas and retinal pigment epithelium differentiation [24, 25], but the mechanism in each process is still unclear. Nicotinamide is widely proclaimed to act through NAD^+^ metabolism, Sirtuin and PARP pathways [40–42]. However, we demonstrate that nicotinamide carries out many functions as a kinase inhibitor in hESCs [22]. The expanded kinase inhibition screen identified 18 nicotinamide targets in 468 kinase candidates (Fig. 3C, D). Among the kinases inhibited by nicotinamide, P38 inhibition leads to cardiac differentiation (Fig. 3F, G), while ROCK inhibition improves cardiomyocyte survival (Fig. 6). These findings highlight nicotinamide’s important regulatory mechanism through kinase cascades, and we believe that more nicotinamide actions will be associated with kinase inhibition.

While WNT inhibition is the most common approach to induce cardiomyocytes from mesoderm progenitors, other pathways are also important for cardiac cell fate determination both in vivo and in vitro [28, 43, 44]. Although P38 inhibition has been previously implicated in promoting cardiomyocyte on the embryoid body platform [45], this study is the first to reveal the stage-specific impact of P38 inhibition in mesodermal differentiation in chemically defined conditions. Beyond the function of cardiac differentiation, P38 has also been implicated in cardiomyocyte proliferation and hypertrophy, as well as sarcomeric organization [46–50]. These studies and our research indicate that nicotinamide and P38 pathway regulate cardiac development and pathology. The hESC-based cardiac differentiation process would be an ideal platform to further elaborate the molecular mechanism of nicotinamide function and its crosstalk with WNT pathway in cardiomyocyte fate determination. At the same time, nicotinamide and p38 inhibition could also serve as a viable option to improve cardiac differentiation in albumin-free and chemically defined culture conditions.

In summary, we show that nicotinamide enhances cardiomyocyte differentiation and subsequent cell handling. Through a 468-kinase inhibition screening, we reveal that nicotinamide induces cardiac differentiation partially through the inhibition of p38 pathway. Nicotinamide is also beneficial in cardiomyocyte manipulation by improving cell survival as a ROCK inhibitor. This study not only demonstrates nicotinamide as a useful factor for cardiomyocyte production in regenerative medicine, but also implicates that nicotinamide could have a profound impact on embryogenesis if it is prescribed during pregnancy.

## Supplementary information

supplemental file

Supplementary Figure 1

Supplementary Figure 2

Supplementary Figure 3

Supplementary Figure 4

Supplementary Figure 5

Supplementary Figure 6

Supplementary Table 1

Supplementary Table 2

Supplementary Video

co-authors’ agreement

## Data Availability

All data in the manuscript and supplementary information are available from the corresponding authors upon request.
