# Supplementary material for: Nicotinamide promotes cardiomyocyte derivation and survival through kinase inhibition in human pluripotent stem cells
Source: Cell Death Dis. 2021 Nov 29;12(12):1119. doi: 10.1038/s41419-021-04395-z (PMC8630224; doi:10.1038/s41419-021-04395-z)
Supplement: Supplementary file 1 — supplemental file [file 41419_2021_4395_MOESM1_ESM.docx]

**Nicotinamide promotes cardiomyocyte derivation and survival through kinase inhibition in human pluripotent stem cells**

**Supplemental information**

Supplementary methods

Supplementary Fig. 1. Nicotinamide directs hPSCs towards cardiomyocytes in mesoderm differentiation.

Supplementary Fig. 2. Field potential of cardiomyocyte induced by IWP-2 or nicotinamide.

Supplementary Fig. 3. Mechanism study of nicotinamide-induced cardiac differentiation.

Supplementary Fig. 4. Comparison of gene expression in cardiomyocytes induced by WNT inhibitor, P38 inhibitor, or nicotinamide.

Supplementary Fig. 5. Nicotinamide promotes the survival of cardiomyocytes.

Supplementary Fig. 6. The effect of nicotinamide-related inhibitors on VEGF-induced endothelial differentiation.

Supplementary Video. The morphology of cardiomyocytes induced by nicotinamide on day 8 of differentiation.

Supplementary Table 1. Reagents and primers used in this study.

Supplementary Table 2. The full results of KINOMEscan assay.

**Supplementary methods**

**Cardiac differentiation in H9 and NL4 cell lines**

H9 and NL4 cells were seeded in matrigel-coated 12-well plates, and cultured for 2 days. CHIR99021 was added in E5 basal medium on day 0 of differentiation. After mesodermal induction by CHIR99021 for 1 day, the cells were cultured in E5 medium for 1 day. Then IWP-2 (3 μM) was applied from day 2 to day 5 as positive control, and no treatment between day 2 and day 5 was used as negative control. Nicotinamide (10 mM) in the presence or absence of IWP-2 was added from day 2 to day 5 to examine the synergistic effect. The optimized method of nicotinamide application was also tested in H9 and NL4 lines. Nicotinamide at 10 mM or 20 mM was applied to H9 or NL4 cells from day 1 to day 5 of differentiation. Total RNA was harvested on day 10 of differentiation for q-PCR.

**Multi-electrode Array (MEA) assay**

The assay was performed following the instruction of manufacture (Axion) with some modification using the MAESTRO 768D system. Briefly, the MEA plate was coated with vitronectin (20 μg/ml) for at least 1 hour at 37°C, 5% CO2. Then cardiomyocytes were dissociated with accutase for 10 minutes at 37°C. After removing accutase, cardiomyocytes were suspended in E5 medium with insulin (10 μg/ml) and Y27632 (10 μM), and the cell suspension was centrifuged at 1000 rpm for 5 minutes. The cell pellet was resuspended in E5 medium with insulin (10 μg/ml) and Y27632 (10 μM) to 8,000 cells /μl. After removing the vitronectin from MEA plate, 8 μl cell suspension was added into the center of each well of 12-well MEA plate, and incubated at 37°C, 5% CO2 for 1-2 hours. Cardiomyocyte Maintenance Medium (Gibco, A2920801) was added gently into each well of MEA plate. After 5-7 days of culture, the electrical signals were recorded on Axion Biosystems’ Maestro MEA system.

**Mito stress test**

Mito stress test was performed using seahorse XF cell mito stress test kit (Agilent, 103015-100) on seahorse XFe 96 extracellular flux analyzer (Agilent). Briefly, IWP-2 or nicotinamide-induced cardiomyocytes were seeded into the seahorse XF cell culture microplates (Agilent, 101085-004) on day 10-15 of differentiation. The cells were cultured in E5 medium with insulin (10 μg/ml) and Y27632 (10 μM) for 1 day. The experiment was performed following the manufacture’s protocol. These reagents were used for Mito Stress test: oligomycin (2 μM), FCCP (1 μM), rotenone/antimycin A (0.5 μM). After the test, cells were lysed in wells (10 mM Tris-HCl, 0.1% triton), and protein level was measured using Bradford reagent for normalization.

**Lentivirus packaging and transfection**

The plasmids of shRNA targeted *ROCK1*, *ROCK2*, *MAPK11*, *MAPK12*, *MAPK13* and *MAPK14* were constructed by GeneCopoeia Company. The forward primers used for plasmid construction were 5’-GATCCG(sense strand)TCAAGAG(antisense strand)TTTTTTGGAATT-3’. The targeted sequences were listed here.

*ROCK1-c* GCAGATATAAAGACACAATAA

*ROCK1-e* GGCGAAATGGTGTAGAAGAAA

*ROCK2-c* GGAACACCGGATTATATATCA

*ROCK2-d* GGATTCACTTGTAGGAACATA

*MAPK11-a* GAATCTACACGCATGTATGCA

*MAPK11-b* TGAACAACATCGTCAAGTGCC

*MAPK12-b* GTCCAGAAGTATGATGACTCC

*MAPK12-c* GATGAATGGAAGCGTGTTACT

*MAPK13-f* GCTGGATGCACTACAACCAGA

*MAPK13-h* GCACATCTACAAGGAGATTGT

*MAPK14-a* CCATTTCAGTCCATCATTCAT

*MAPK14-b* CCTAGTAATCTAGCTGTGAAT

The shRNA plasmid was transfected together with psPAX2 and pMD2.G into 293FT cells. After transfection, the medium containing virus was harvested and precipitated by Polyethylene glycol (PEG, sigma). The virus was suspended in PBS, and aliquoted for transfection. To determine the knockdown efficiency, H1 cells were transfected with shRNA lentivirus, and total RNA was harvested for q-PCR. The efficient lentivirus was used to knock down gene expression during cardiac differentiation. The indicated virus was added into the E5 medium on day 2 of differentiation, and RNA was harvested to detect the mRNA level of *NKX2-5* on day 10 of differentiation.

**Cardiomyocyte passaging and immunoblot analysis**

The cardiomyocytes were dissociated by TrypLE for 10 minutes on day 10-13 of differentiation. After removing of TrypLE, the cardiomyocytes were suspended in E5 basal medium with 10 μg/ml insulin, and seeded into the Matrigel-coated plates. After 1 hour, the cells were harvested for western blot using 2 x loading buffer included protease and phosphatase Inhibitors. The cell number of cardiomyocytes were counted with flow cytometry after 24 hours of seeding.

After dissociation, the cardiomyocytes were seeded onto the Matrigel-coated plates, and treated with the indicated chemicals for 24 hours before the cells were harvested for western blot. Two primary antibodies were used: LC3 (1:1000, Cell signaling, 4599s) and P62 (1:2000, MBL, PM045). The protein expression levels were quantified by Image Lab (Biorad).

**VEGF-induced differentiation**

H1 cells were seeded in Matrigel-coated 12-well plates, and cultured for 2 days. CHIR99021 (5 μM) was added in E5 basal medium on day 0 of differentiation. After mesodermal induction by CHIR99021 for 1 day, the cells were cultured in E5 medium for 1 day. Then 50 ng/ml VEGF was added into E5 basal medium with or without the following drugs: Nicotinamide (Nam, 10 mM), P38 inhibitor SB202190 (P38i, 10 μM), ROCK inhibitor Y-27632 (ROCKi, 10 μM), CSNK1 inhibitor D4476 (CSNK1i, 10 μM), MEK5 inhibitor BIX02189 (MEK5i, 5 μM), PARP inhibitor AZD2281 (PARPi, 100 nM), SIRT1 inhibitor EX527 (SIRT1i, 10 μM), or Niacin (10 mM). After treated for 3 days, the cells were cultured for 4 days in E5 medium, and then total RNA was harvested to measure the gene expression of *PDGFRβ*, *ACTA2*, *PECAM* and *CDH5* at day 9 of differentiation.

**Supplementary Fig. 1.** Nicotinamide directs hPSCs towards cardiomyocytes in mesoderm differentiation.

**A-B** The gene ontology (GO) analysis showing the biological processes related to development and differentiation regulated by nicotinamide (Nam). H1 cells were treated with 10 mM nicotinamide for 3 days, and then the samples were harvested for microarray. The genes upregulated by nicotinamide **(A)** were enriched in the 9 development-related processes (*p < 0.05). Panel **B** shows the top 20 development-related biological processes enriched by the downregulated genes in nicotinamide group.

**C-D** Analyses of mRNA expression levels of *NKX2-5* and *TNNT2* at day 10 of cardiac differentiation in H9 hESC (**C**) and NL4 iPSC (**D**). Control, no treatment from day 2 to day 5. The following treatments were added from day 2 to day 5, respectively. IWP-2 (3 μM); Nicotinamide (Nam, 10mM); IWP-2 and nicotinamide (IWP-2+Nam). Data shown are mean ± SD of 3 independent experiments (*p < 0.05 compared with control, #p < 0.05 compared with IWP-2).

**E-F** The mRNA levels of cardiac marker genes *NKX2-5* and *TNNT2* were measured at day 10 of differentiation in hESC H9 (**E**) and iPSC NL4 (**F**). IWP-2 (3 μM) was added from day 2 to day 5, and nicotinamide at 10 mM or 20 mM was added from day 1 to day 5. Data shown are mean ± SD of 3 independent experiments (*p < 0.05 compared with control).

**Supplementary Fig. 2.** Functional analysis of IWP-2 or nicotinamide-derived cardiomyocytes using multielectrode array (MEA) system.

**A** The representative waveform of IWP-2 or nicotinamide-derived cardiomyocytes was recorded using Axion Biosystems. The IWP-2 or nicotinamide-derived cardiomyocytes were seeded in the 12-well MEA plates on day 15 of differentiation, and the electrical signals were recorded on day 25.

**B** The beat period, conduction velocity, and maximum propagation delay were measured in IWP-2 or nicotinamide-derived cardiomyocytes by MEA. Data shown are mean ± SD of 14-28 measurements (*p < 0.05 compared with IWP-2).

**Supplementary Fig. 3.** Mechanism study of nicotinamide-induced cardiac differentiation.

**A-B** The effect of HDAC inhibitor VPA on cardiac differentiation. Different doses of VPA was added from day 1 to day 5 of differentiation. IWP-2 (3 μM) was added from day 2 to day 5 (IWP-2), and nicotinamide at 10 mM or 20 mM was added from day 1 to day 5.

**C-D** The gene expression of *ROCK1* (**C**) and *ROCK2* (**D**) was quantified by q-PCR. H1 cells were transfected with the indicated shRNA lentivirus targeted *ROCK1* or *ROCK2* for 24 hours, and total RNA was harvested after 48 hours for q-PCR.

**E-H** The gene expression of *MAPK11* (**E**), *MAPK12* (**F**), *MAPK13* (**G**), and *MAPK14* (**H**) was quantified by q-PCR. H1 cells were transfected with the indicated shRNA lentivirus targeted *MAPK11* (**E**), *MAPK12* (**F**), *MAPK13* (**G**), or *MAPK14* (**H**) for 24 hours, and total RNA was harvested after 48 hours for q-PCR.

**I** The gene expression of *NKX2-5* was analyzed by q-PCR. On day 0, CHIR99021 was added to the E5 differentiation medium for 24 hours. Then the cells were cultured in E5 medium for 1 day. On day 2, the efficient shRNA lentivirus were added to infect the cells for 1 day. After infection, the cells were cultured in E5 medium for 7 days, and total RNA was harvested for q-PCR on day 10 of differentiation. Data shown are mean ± SD of 3 independent experiments (*p < 0.05 compared with Control or sh-Control).

**Supplementary Fig. 4.** Comparison of gene expression in cardiomyocytes induced by WNT inhibitor, P38 inhibitor, or nicotinamide.

**A** The top 10 pathways enriched by the 644 genes specifically upregulated by nicotinamide (Nam), SB202190 (P38i), and IWP-2 treatment.

**B** The top 10 pathways enriched by the 562 genes specifically downregulated by nicotinamide (Nam), SB202190 (P38i), and IWP-2 treatment.

**C** The top 10 pathways enriched by the 499 genes specifically upregulated by nicotinamide, but not IWP-2 or P38 inhibitor.

**D** The top 10 pathways enriched by the 494 genes specifically downregulated by nicotinamide, but not IWP-2 or P38 inhibitor.

**E** Gene expressions related to electron transport in the differentiated cells after 10 days of differentiation.

**Supplementary Fig. 5.** Nicotinamide promotes the survival of cardiomyocytes.

**A** Immunoblot analysis of p-MLC / MLC in Fig. 6C. Data shown are mean ± SD of 3 independent experiments (*p < 0.05 compared with Control).

**B-E** hESC-derived cardiomyocytes were passaged and subjected to the indicated treatments for 24 hours. The protein levels of LC3 and p62 were determined by western blot. Chloroquine increased the protein level of LC3-II and P62 significantly. Neither nicotinamide nor ROCK inhibitor Y27632 show significant impact on the LC3-II and P62 in the condition with or without leupetin and chloroquine treatment. Data shown are mean ± SD of 3 independent experiments (*p < 0.05 compared with none treated control).
**F** Dose-dependent effect of nicotinamide on the survival of cardiomyocytes generated through nicotinamide. After 10 days of differentiation, hESC-derived cardiomyocytes by nicotinamide were passaged under the indicated doses of nicotinamide (Nam) or Y27632 10 μM (ROCKi). The survival index indicates the number of living cells divided by the input cells 24 hours after seeding (n = 3 technical repeats). Data shown are representative of 3 independent experiments (*p < 0.05 compared with Control).

**G** Representative phase contrast images of cardiomyocytes induced by nicotinamide before and 24 hours after seeding. Scale bar, 100 μm.

**Supplementary Fig. 6.** The effect of nicotinamide-related inhibitors on VEGF-induced endothelial differentiation.

**A-B** The indicated inhibitors were added to analyze the mechanism of nicotinamide in VEGF-induced differentiation. The mRNA levels of *PECAM* and *CDH5* were measured by q-PCR at day 9 of differentiation. The following treatments were applied from day 2 to day 5 together with VEGF 50 ng/mL, respectively. Nicotinamide (Nam, 10 mM), P38 inhibitor SB202190 (10 μM), ROCK inhibitor Y-27632 (10 μM), CK1 inhibitor D4476 (10 μM), MEK5 inhibitor BIX02189 (5 μM), PARP inhibitor AZD2281 (100 nM), Sirt1 inhibitor EX527 (10 μM), Niacin (10 mM).
