# Supplementary figures and images for: Nicotinamide promotes cardiomyocyte derivation and survival through kinase inhibition in human pluripotent stem cells
Source: Cell Death Dis. 2021 Nov 29;12(12):1119. doi: 10.1038/s41419-021-04395-z (PMC8630224; doi:10.1038/s41419-021-04395-z)

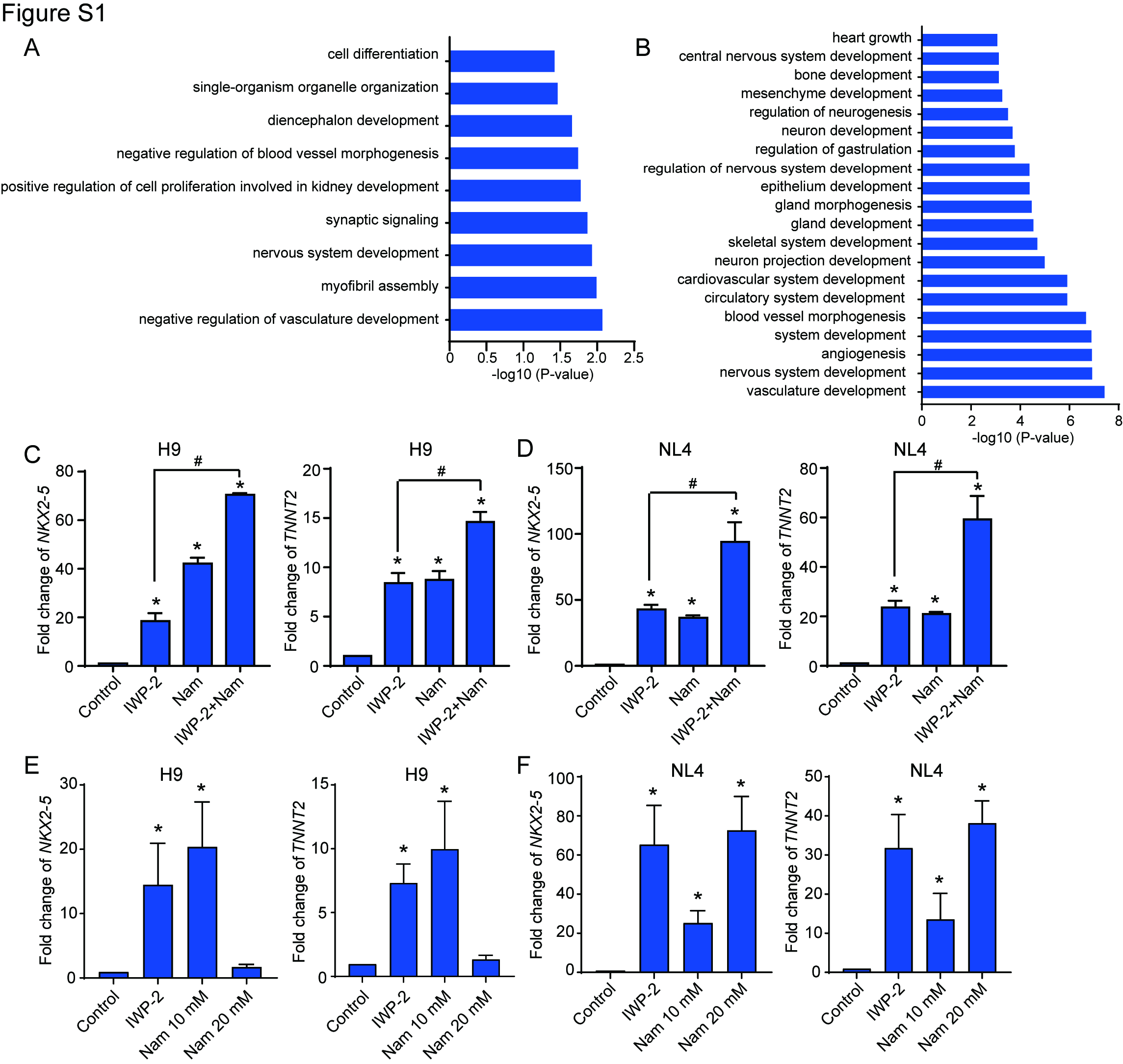

Supplement: Supplementary file 2 — Supplementary Figure 1 [file 41419_2021_4395_MOESM2_ESM.tif]

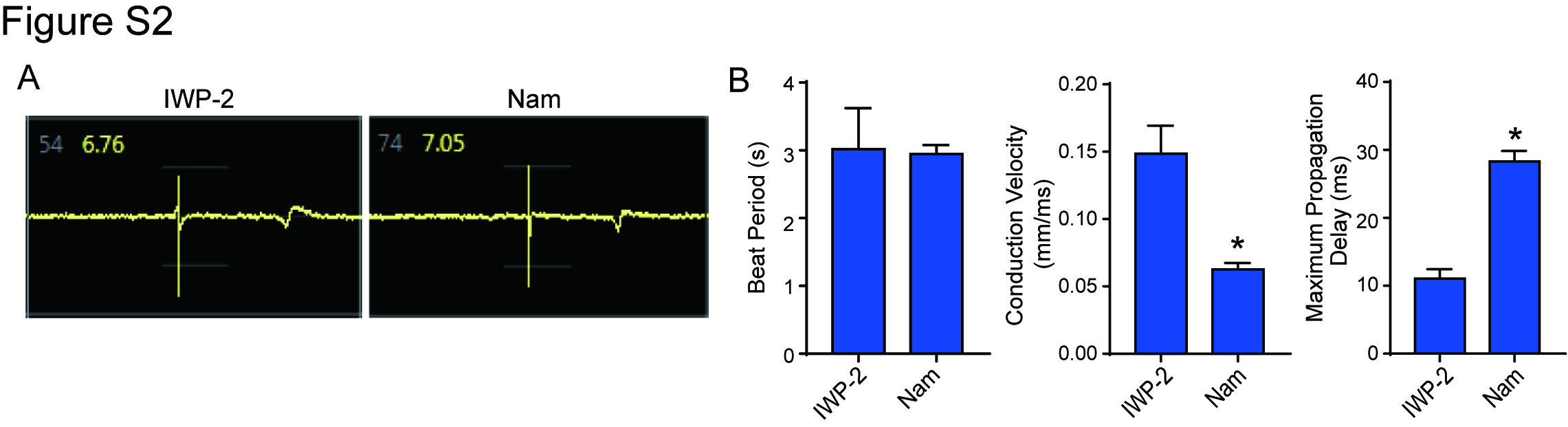

Supplement: Supplementary file 3 — Supplementary Figure 2 [file 41419_2021_4395_MOESM3_ESM.tif]

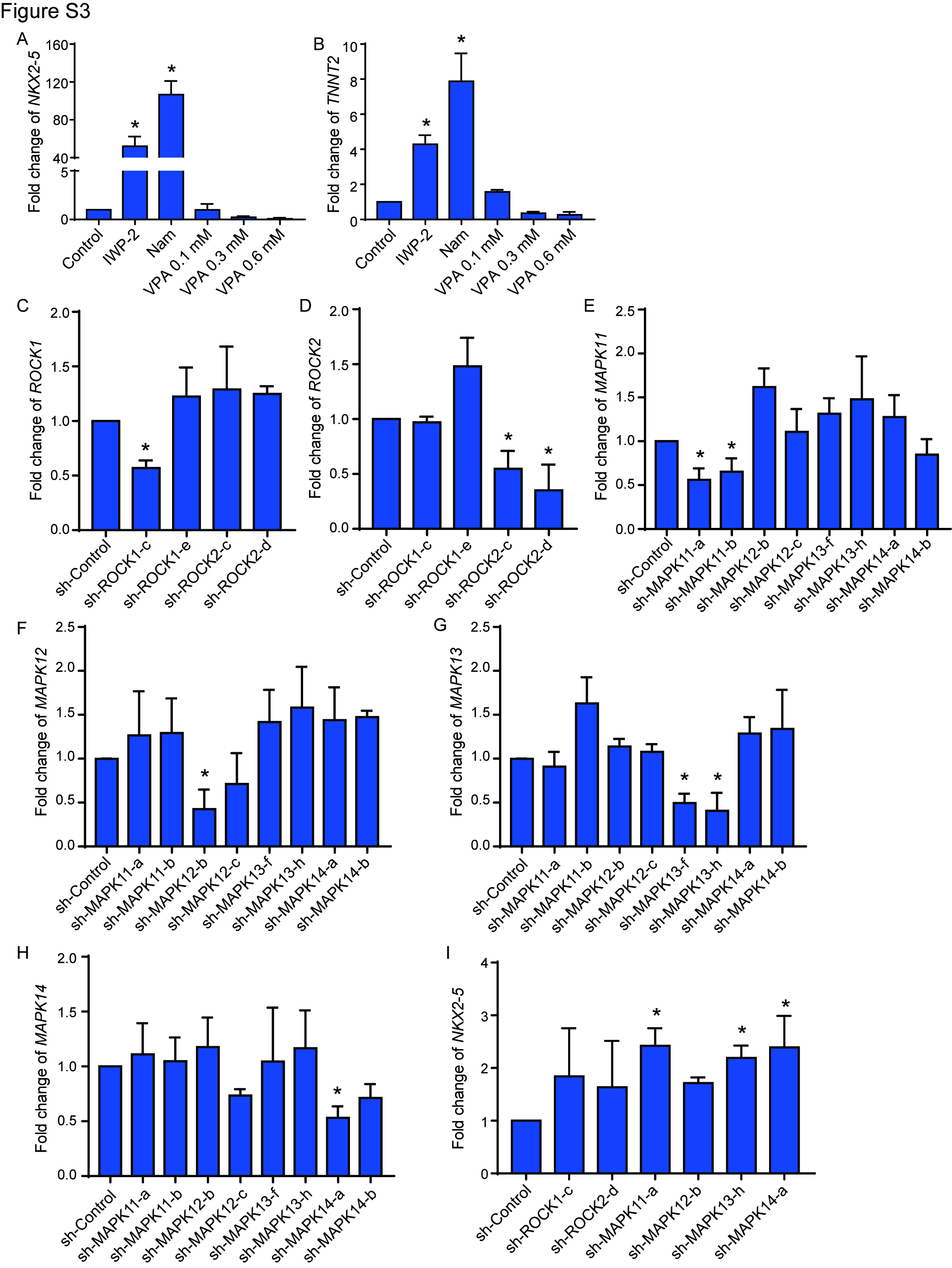

Supplement: Supplementary file 4 — Supplementary Figure 3 [file 41419_2021_4395_MOESM4_ESM.tif]

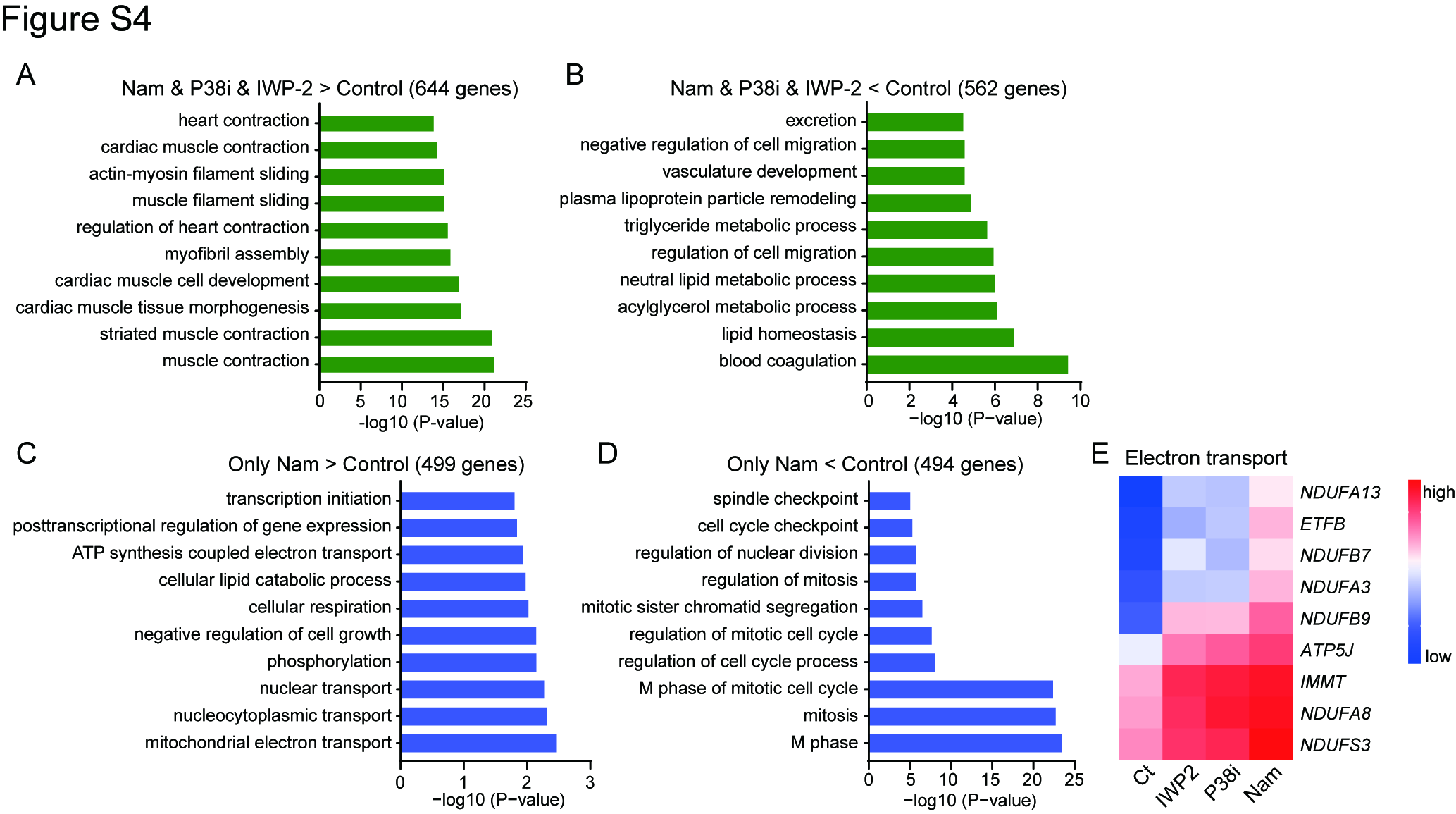

Supplement: Supplementary file 5 — Supplementary Figure 4 [file 41419_2021_4395_MOESM5_ESM.tif]

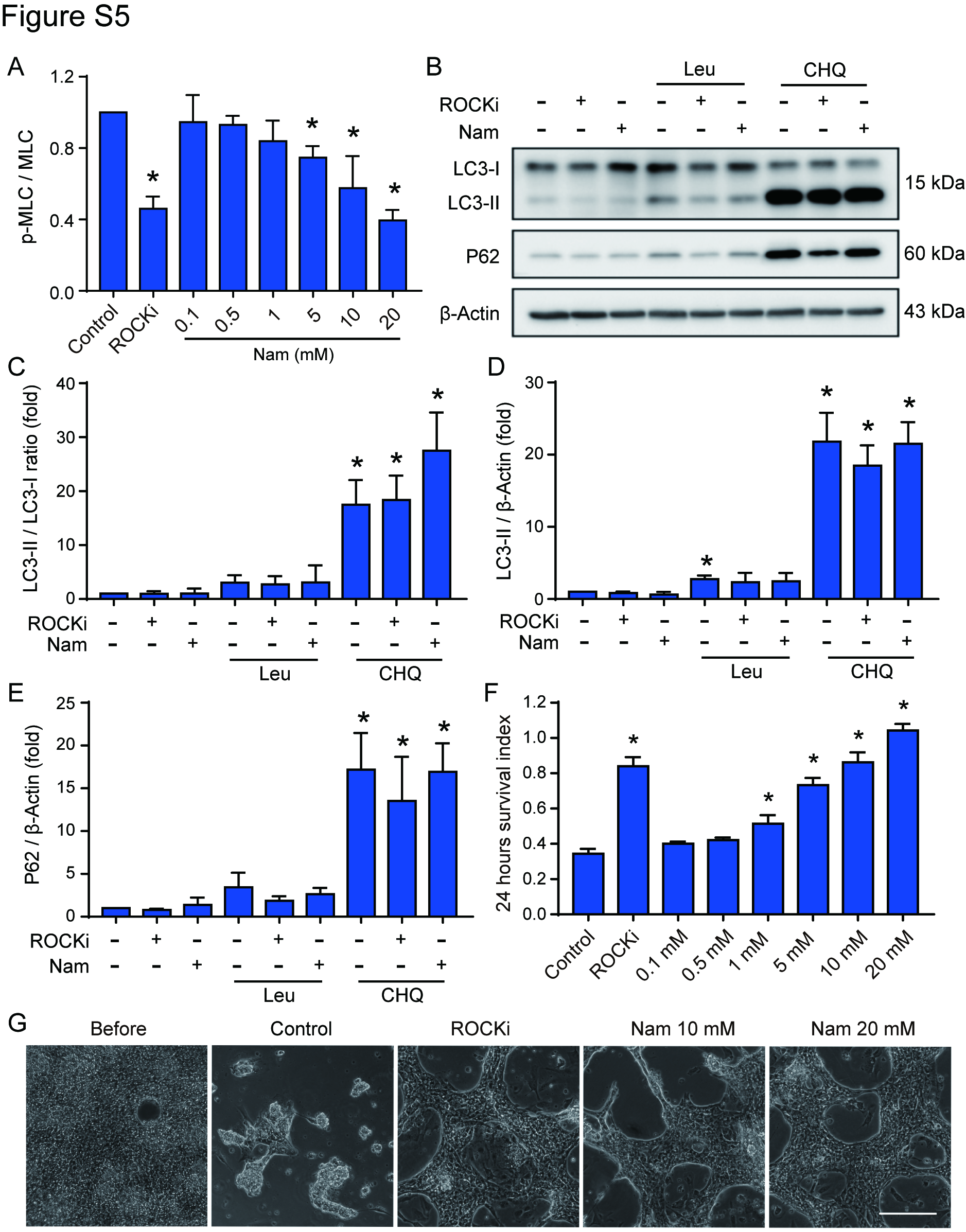

Supplement: Supplementary file 6 — Supplementary Figure 5 [file 41419_2021_4395_MOESM6_ESM.tif]

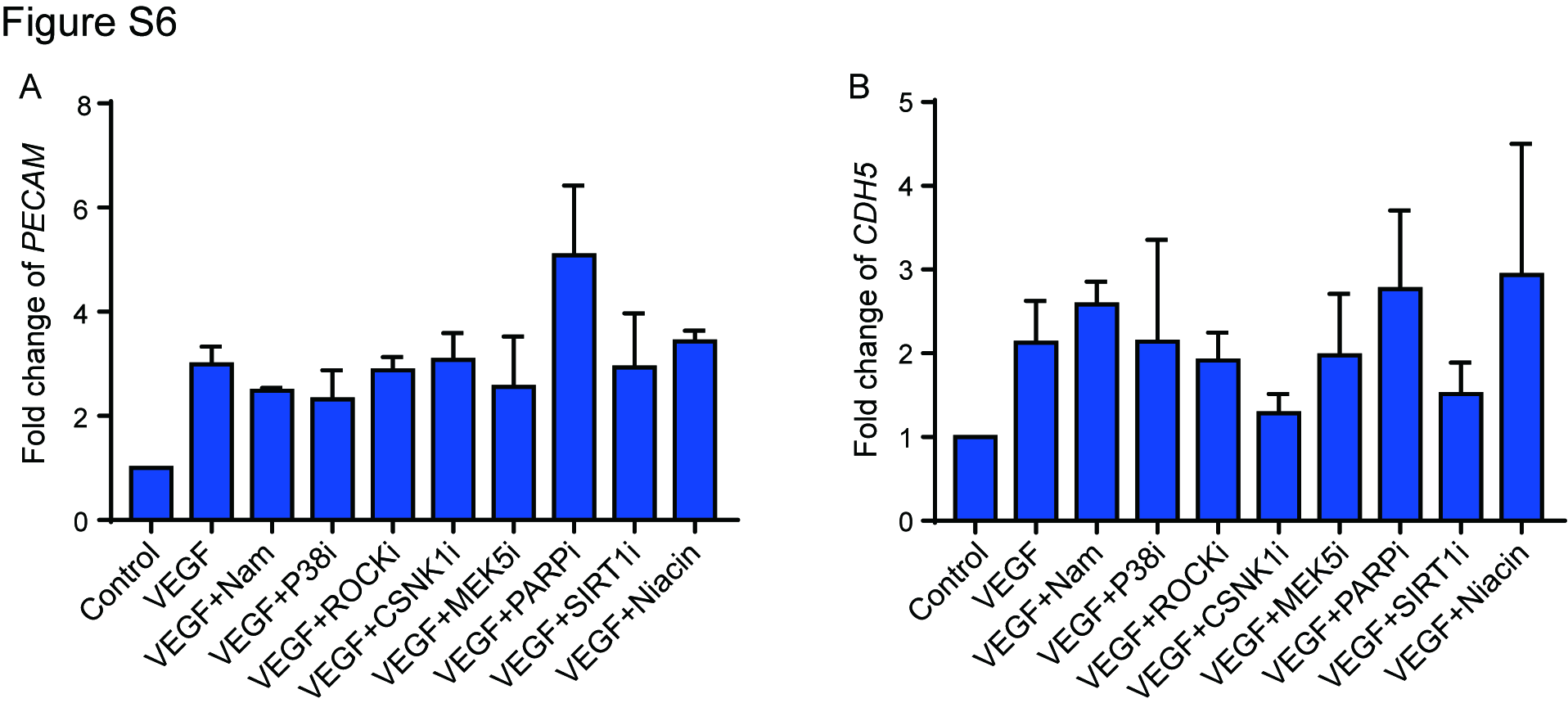

Supplement: Supplementary file 7 — Supplementary Figure 6 [file 41419_2021_4395_MOESM7_ESM.tif]
