# Supplementary material for: Nicotinamide promotes cardiomyocyte derivation and survival through kinase inhibition in human pluripotent stem cells
Source: Cell Death Dis. 2021 Nov 29;12(12):1119. doi: 10.1038/s41419-021-04395-z (PMC8630224; doi:10.1038/s41419-021-04395-z)
Supplement: Supplementary file 11 — co-authors’ agreement [file 41419_2021_4395_MOESM11_ESM.pdf]

Re:author change

"孟雅" <mengya_85@163.com>
收件人: mengya_85@163.com
时 间: 2021-10-26 15:59:42
附 件:

I am OK with the changes. Thank you!

Best regards,

Ya Meng

At 2021-10-26 15:28:44, "孟雅" <mengya_85@163.com> wrote:

> Dear Dr. Meng,
>
> Thank you for your contribution to the study “Nicotinamide promotes cardiomyocyte derivation and survival through kinase inhibition in human pluripotent stem cells". It has been accepted for publication in Cell Death & Disease.
>
> During the review process, Dr. Chon Lok LEI helped analysis electrophysiology data, so we added him as a co-author. Meanwhile, Dr. Chengcheng Song conducted significant amount wetlab experiments to address reviewers' concerns, so we adjusted her position in the author list. The author lists are below.
>
> The author list in original manuscript: Ya Meng, Zhili Ren, Chengcheng Song, Xiaohong Li, Xiangyu Yang, Nana Ai, Yang Yang, Dongjin Wang, Meixiao Zhan, Jiaxian Wang, Weiwei Liu, Wei Ge, Ligong Lu*, and Guokai Chen*.
>
> The author list in current manuscript: Ya Meng, Chengcheng Song, Zhili Ren, Xiaohong Li, Xiangyu Yang, Nana Ai, Yang Yang, Dongjin Wang, Meixiao Zhan, Jiaxian Wang, Chon Lok LEI, Weiwei Liu, Wei Ge, ligong lu*, and Guokai Chen*.
>
> Please reply this email to confirm whether you are OK with the changes. Thanks for your cooperation.
>
> Best regards,
>
> Guokai Chen & Ya Meng

答复: author change

chengchengsong <chengchengsong@um.edu.mo>

收件人: "孟雅" <mengya_85@163.com>

时　间: 2021-10-27 9:30:07

附　件:

Dear Dr. Meng

I'm OK for that. thanks for your notice.

Regards,

Chengcheng

发件人: 孟雅 <mengya_85@163.com>

发送时间: 2021年10月26日 15:30:49

收件人: chengchengsong

抄送: guokaichen

主题: author change

Dear Dr. Song,

Thank you for your contribution to the study “Nicotinamide promotes cardiomyocyte derivation and survival through kinase inhibition in human pluripotent stem cells". It has been accepted for publication in Cell Death & Disease.

During the review process, Dr. Chon Lok LEI helped analysis electrophysiology data, so we added him as a co-author. Meanwhile, Dr. Chengcheng Song conducted significant amount wetlab experiments to address reviewers' concerns, so we adjusted her position in the author list. The author lists are below.

The author list in original manuscript: Ya Meng, Zhili Ren, Chengcheng Song, Xiaohong Li, Xiangyu Yang, Nana Ai, Yang Yang, Dongjin Wang, Meixiao Zhan, Jiaxian Wang, Weiwei Liu, Wei Ge, Ligong Lu*, and Guokai Chen*.
The author list in current manuscript: Ya Meng, Chengcheng Song, Zhili Ren, Xiaohong Li, Xiangyu Yang, Nana Ai, Yang Yang, Dongjin Wang, Meixiao Zhan, Jiaxian Wang, Chon Lok LEI, Weiwei Liu, Wei Ge, ligong lu*, and Guokai Chen*.

Please reply this email to confirm whether you are OK with the changes. Thanks for your cooperation.

Best regards,

Guokai Chen & Ya Meng

Re: author change

"ZHILI REN" <ZHILIREN@outlook.com>

收件人: "孟雅" <mengya_85@163.com>

抄 送: "guokaichen@umac.mo" <guokaichen@umac.mo>

时 间: 2021-10-26 17:30:27

附 件:

Hi Ya,

Thank you for letting me know. I can confirm that I agree with the changes. Thanks!

Best wishes,
Zhili

From: 孟雅 <mengya_85@163.com>
Sent: 26 October 2021 8:50
To: zhiliren@outlook.com <zhiliren@outlook.com>
Cc: guokaichen@umac.mo <guokaichen@umac.mo>
Subject: author change

Dear Dr. Ren,

Thank you for your contribution to the study "Nicotinamide promotes cardiomyocyte derivation and survival through kinase inhibition in human pluripotent stem cells". It has been accepted for publication in Cell Death & Disease.

During the review process, Dr. Chon Lok LEI helped analysis electrophysiology data, so we added him as a co-author. Meanwhile, Dr. Chengcheng Song conducted significant amount wetlab experiments to address reviewers' concerns, so we adjusted her position in the author list. The author lists are below.

The author list in original manuscript: Ya Meng, Zhili Ren, Chengcheng Song, Xiaohong Li, Xiangyu Yang, Nana Ai, Yang Yang, Dongjin Wang, Meixiao Zhan, Jiaxian Wang, Weiwei Liu, Wei Ge, Ligong Lu*, and Guokai Chen*.
The author list in current manuscript: Ya Meng, Chengcheng Song, Zhili Ren, Xiaohong Li, Xiangyu Yang, Nana Ai, Yang Yang, Dongjin Wang, Meixiao Zhan, Jiaxian Wang, Chon Lok LEI, Weiwei Liu, Wei Ge, ligong lu*, and Guokai Chen*.
Please reply this email to confirm whether you are OK with the changes. Thanks for your cooperation.

Best regards,

Guokai Chen & Ya Meng

Re:author change

"李晓红" <lixiaohong@gdph.org.cn>

收件人: "孟雅" <mengya_85@163.com>

时　间: 2021-10-27 10:50:30

附　件:

Dear Guokai Chen & Ya Meng,

I totally agree the change of author list. Thanks for your cooperation in this study.

Beat regards,

Xiaohong Li

李晓红

广东省人民医院（广东省医学科学院）/广东省华南结构性心脏病重点实验室

13802801520

------------------ Original ------------------

From: "孟雅"<mengya_85@163.com>;

Date: Tue, Oct 26, 2021 03:32 PM

To: "李晓红"<lixiaohong@gdph.org.cn>;

Cc: "guokaichen"<guokaichen@umac.mo>;

Subject: author change

Dear Prof. Li,

Thank you for your contribution to the study “Nicotinamide promotes cardiomyocyte derivation and survival through kinase inhibition in human pluripotent stem cells". It has been accepted for publication in Cell Death & Disease.

During the review process, Dr. Chon Lok LEI helped analysis electrophysiology data, so we added him as a co-author. Meanwhile, Dr. Chengcheng Song conducted significant amount wetlab experiments to address reviewers' concerns, so we adjusted her position in the author list. The author lists are below.

The author list in original manuscript: Ya Meng, Zhili Ren, Chengcheng Song, Xiaohong Li, Xiangyu Yang, Nana Ai, Yang Yang, Dongjin Wang, Meixiao Zhan, Jiaxian Wang, Weiwei Liu, Wei Ge, Ligong Lu*, and Guokai Chen*.

The author list in current manuscript: Ya Meng, Chengcheng Song, Zhili Ren, Xiaohong Li, Xiangyu Yang, Nana Ai, Yang Yang, Dongjin Wang, Meixiao Zhan, Jiaxian Wang, Chon Lok LEI, Weiwei Liu, Wei Ge, ligong lu*, and Guokai Chen*.

Please reply this email to confirm whether you are OK with the changes. Thanks for your cooperation.

Best regards,

Guokai Chen & Ya Meng

Re: author change

yangxygdzhh <yangxygdzhh@163.com>

收件人: "孟雅" <mengya_85@163.com>

抄　送: guokaichen <guokaichen@umac.mo>

时　间: 2021-10-27 13:57:27

附　件:

Hi Ya,

Thank you for letting me know. I can confirm that I agree with the changes. Thanks!

Best wishes,
Xiangyu

杨翔宇
邮箱: yangxygdzhh@163.com

签名由 网易邮箱大师 定制

On 10/26/2021 15:34, 孟雅 wrote:

Dear Dr. Yang,

Thank you for your contribution to the study "Nicotinamide promotes cardiomyocyte derivation and survival through kinase inhibition in human pluripotent stem cells". It has been accepted for publication in Cell Death & Disease.

During the review process, Dr. Chon Lok LEI helped analysis electrophysiology data, so we added him as a co-author. Meanwhile, Dr. Chengcheng Song conducted significant amount wetlab experiments to address reviewers' concerns, so we adjusted her position in the author list. The author lists are below.

The author list in original manuscript: Ya Meng, Zhili Ren, Chengcheng Song, Xiaohong Li, Xiangyu Yang, Nana Ai, Yang Yang, Dongjin Wang, Meixiao Zhan, Jiaxian Wang, Weiwei Liu, Wei Ge, Ligong Lu*, and Guokai Chen*.

The author list in current manuscript: Ya Meng, Chengcheng Song, Zhili Ren, Xiaohong Li, Xiangyu Yang, Nana Ai, Yang Yang, Dongjin Wang, Meixiao Zhan, Jiaxian Wang, Chon Lok LEI, Weiwei Liu, Wei Ge, ligong lu*, and Guokai Chen*.

Please reply this email to confirm whether you are OK with the changes. Thanks for your cooperation.

Best regards,

Guokai Chen & Ya Meng

RE: author change

nanaai <nanaai@um.edu.mo>

收件人: "孟雅" <mengya_85@163.com>

抄　送: guokaichen <guokaichen@um.edu.mo>

时　间: 2021-10-26 15:40:23

附　件:

Dear Mengya,

It's ok. Thank you!

Best regards,

Nana AI

Room 2036, N22 Building,

Faculty of Health Sciences (FHS),

University of Macau,

Avenida de Universidade, Taipa,

Macau SAR, China

E-mail: nanaai@um.edu.mo

Tel: (853) 8822 2735

寄件者: 孟雅 <mengya_85@163.com>

寄件日期: 2021年10月26日 15:34

收件者: nanaai

副本: guokaichen

主旨: author change

Dear Dr. Ai,

Thank you for your contribution to the study “Nicotinamide promotes cardiomyocyte derivation and survival through kinase inhibition in human pluripotent stem cells". It has been accepted for publication in Cell Death & Disease.

During the review process, Dr. Chon Lok LEI helped analysis electrophysiology data, so we added him as a co-author. Meanwhile, Dr. Chengcheng Song conducted significant amount wetlab experiments to address reviewers' concerns, so we adjusted her position in the author list. The author lists are below.

The author list in original manuscript: Ya Meng, Zhili Ren, Chengcheng Song, Xiaohong Li, Xiangyu Yang, Nana Ai, Yang Yang, Dongjin Wang, Meixiao Zhan, Jiaxian Wang, Weiwei Liu, Wei Ge, Ligong Lu*, and Guokai Chen*.

The author list in current manuscript: Ya Meng, Chengcheng Song, Zhili Ren, Xiaohong Li, Xiangyu Yang, Nana Ai, Yang Yang, Dongjin Wang, Meixiao Zhan, Jiaxian Wang, Chon Lok LEI, Weiwei Liu, Wei Ge, ligong lu*, and Guokai Chen*.

Please reply this email to confirm whether you are OK with the changes. Thanks for your cooperation.

Best regards,

Guokai Chen & Ya Meng

回复: author change

"Yang Yang" <yb47624@connect.um.edu.mo>

收件人: "孟雅" <mengya_85@163.com>

抄　送: guokaichen <guokaichen@um.edu.mo>

时　间: 2021-10-27 12:46:43

附　件:

Dear Dr. Meng, Prof. Chen,

I'm OK with the changes, thanks!

Yang Yang

发件人: 孟雅 <mengya_85@163.com>
发送时间: 2021年10月26日 15:36
收件人: Yang Yang <yb47624@connect.um.edu.mo>
抄送: guokaichen <guokaichen@um.edu.mo>
主题: author change

Dear Dr. Yang,

Thank you for your contribution to the study “Nicotinamide promotes cardiomyocyte derivation and survival through kinase inhibition in human pluripotent stem cells". It has been accepted for publication in Cell Death & Disease.

During the review process, Dr. Chon Lok LEI helped analysis electrophysiology data, so we added him as a co-author. Meanwhile, Dr. Chengcheng Song conducted significant amount wetlab experiments to address reviewers' concerns, so we adjusted her position in the author list. The author lists are below.

The author list in original manuscript: Ya Meng, Zhili Ren, Chengcheng Song, Xiaohong Li, Xiangyu Yang, Nana Ai, Yang Yang, Dongjin Wang, Meixiao Zhan, Jiaxian Wang, Weiwei Liu, Wei Ge, Ligong Lu*, and Guokai Chen*.
The author list in current manuscript: Ya Meng, Chengcheng Song, Zhili Ren, Xiaohong Li, Xiangyu Yang, Nana Ai, Yang Yang, Dongjin Wang, Meixiao Zhan, Jiaxian Wang, Chon Lok LEI, Weiwei Liu, Wei Ge, ligong lu*, and Guokai Chen*.
Please reply this email to confirm whether you are OK with the changes. Thanks for your cooperation.

Best regards,

Guokai Chen & Ya Meng

Re: author change

wangdongjin <wangdongjin@njglyy.com>

收件人: "孟雅" <mengya_85@163.com>

时　间: 2021-10-27 15:02:26

附　件:

Dear Ya Meng,
Thanks for your email. I agree with the change.

wangdongjin
邮箱：wangdongjin@njglyy.com

---- Replied Message ----

From	孟雅<mengya_85@163.com>
Date	10/26/2021 15:36
To	wangdongjin@njglyy.com<wangdongjin@njglyy.com>
Cc	guokaichen@umac.mo<guokaichen@umac.mo>
Subject	author change

Dear Dr. Wang,

Thank you for your contribution to the study "Nicotinamide promotes cardiomyocyte derivation and survival through kinase inhibition in human pluripotent stem cells". It has been accepted for publication in Cell Death & Disease.

During the review process, Dr. Chon Lok LEI helped analysis electrophysiology data, so we added him as a co-author. Meanwhile, Dr. Chengcheng Song conducted significant amount wetlab experiments to address reviewers' concerns, so we adjusted her position in the author list. The author lists are below.

The author list in original manuscript: Ya Meng, Zhili Ren, Chengcheng Song, Xiaohong Li, Xiangyu Yang, Nana Ai, Yang Yang, Dongjin Wang, Meixiao Zhan, Jiaxian Wang, Weiwei Liu, Wei Ge, Ligong Lu*, and Guokai Chen*.

The author list in current manuscript: Ya Meng, Chengcheng Song, Zhili Ren, Xiaohong Li, Xiangyu Yang, Nana Ai, Yang Yang, Dongjin Wang, Meixiao Zhan, Jiaxian Wang, Chon Lok LEI, Weiwei Liu, Wei Ge, ligong lu*, and Guokai Chen*.

Please reply this email to confirm whether you are OK with the changes. Thanks for your cooperation.

Best regards,

Guokai Chen & Ya Meng

Re:author change

"Meixiao Zhan" <zhanmeixiao1987@126.com>
收件人: "孟雅" <mengya_85@163.com>
抄 送: guokaichen@umac.mo
时 间: 2021-10-26 21:49:21
附 件:

I am OK with the changes.
Thanks

Meixiao Zhan

At 2021-10-26 15:37:47, "孟雅" <mengya_85@163.com> wrote:

> Dear Prof. Zhan,
>
> Thank you for your contribution to the study “Nicotinamide promotes cardiomyocyte derivation and survival through kinase inhibition in human pluripotent stem cells". It has been accepted for publication in Cell Death & Disease.
>
> During the review process, Dr. Chon Lok LEI helped analysis electrophysiology data, so we added him as a co-author. Meanwhile, Dr. Chengcheng Song conducted significant amount wetlab experiments to address reviewers' concerns, so we adjusted her position in the author list. The author lists are below.
>
> The author list in original manuscript: Ya Meng, Zhili Ren, Chengcheng Song, Xiaohong Li, Xiangyu Yang, Nana Ai, Yang Yang, Dongjin Wang, Meixiao Zhan, Jiaxian Wang, Weiwei Liu, Wei Ge, Ligong Lu*, and Guokai Chen*.
>
> The author list in current manuscript: Ya Meng, Chengcheng Song, Zhili Ren, Xiaohong Li, Xiangyu Yang, Nana Ai, Yang Yang, Dongjin Wang, Meixiao Zhan, Jiaxian Wang, Chon Lok LEI, Weiwei Liu, Wei Ge, ligong lu*, and Guokai Chen*.
>
> Please reply this email to confirm whether you are OK with the changes. Thanks for your cooperation.
>
> Best regards,
>
> Guokai Chen & Ya Meng

Re:author change

"王嘉显" <wangjx@helptx.com.cn>

收件人: "孟雅" <mengya_85@163.com>

抄 送: guokaichen <guokaichen@umac.mo>

时 间: 2021-10-26 18:11:42

附 件:

Dear Mengya,
Congrats! I'm totally fine with the correction.
Best
Jiaxian

Many Thanks & Best Regards

王嘉显 Jiaxian, Eugene Wang

HELP Stem Cell Innovation, President

公司网址: www.helpsci.com.cn

E-mail: wangjx@helpsci.com.cn

Help STEM CELL INNOVATION

本邮件及其附件含有艾尔普公司的保密信息，仅限于发送给上面地址中列出的个人或群组。禁止任何其他人以任何形式使用（包括但不限于全部或部分地泄露、复制、或散发）本邮件中的信息。如果您错收了本邮件，请您立即电话或邮件通知发件人并删除本邮件！

This e-mail and its attachments contain confidential information from HELP, which is intended only for the person or entity whose address is listed above. Any use of the information contained herein in any way(including, but not limited to, total or partial disclosure, reproduction, or dissemination) by persons other than the intended recipient(s) is prohibited. If you receive this e-mail in error, please notify the sender by phone or email immediately and delete it!

------------------ Original ------------------

From: "孟雅"<mengya_85@163.com>;
Date: Tue, Oct 26, 2021 03:38 PM
To: "wangjx"<wangjx@helptx.com.cn>;
Cc: "guokaichen"<guokaichen@umac.mo>;
Subject: author change

Dear Dr. Wang,

Thank you for your contribution to the study “Nicotinamide promotes cardiomyocyte derivation and survival through kinase inhibition in human pluripotent stem cells". It has been accepted for publication in Cell Death & Disease.

During the review process, Dr. Chon Lok LEI helped analysis electrophysiology data, so we added him as a co-author. Meanwhile, Dr. Chengcheng Song conducted significant amount wetlab experiments to address reviewers' concerns, so we adjusted her position in the author list. The author lists are below.

The author list in original manuscript: Ya Meng, Zhili Ren, Chengcheng Song, Xiaohong Li, Xiangyu Yang, Nana Ai, Yang Yang, Dongjin Wang, Meixiao Zhan, Jiaxian Wang, Weiwei Liu, Wei Ge, Ligong Lu*, and Guokai Chen*.

The author list in current manuscript: Ya Meng, Chengcheng Song, Zhili Ren, Xiaohong Li, Xiangyu Yang, Nana Ai, Yang Yang, Dongjin Wang, Meixiao Zhan, Jiaxian Wang, Chon Lok LEI, Weiwei Liu, Wei Ge, ligong lu*, and Guokai Chen*.

Please reply this email to confirm whether you are OK with the changes. Thanks for your cooperation.

Best regards,

Guokai Chen & Ya Meng

Re: author change

chonloklei <chonloklei@um.edu.mo>
收件人："孟雅" <mengya_85@163.com>
抄　送：guokaichen <guokaichen@um.edu.mo>
时　间：2021-10-26 16:38:40
附　件：

Dear Ya,

Congratulations to getting the paper accepted! Yep, that's fine by me, thanks for letting me know.

Best,
Jack

From: 孟雅 <mengya_85@163.com>
Sent: 26 October 2021 15:39:45
To: chonloklei
Cc: guokaichen
Subject: author change

Dear Prof. Lei,

Thank you for your contribution to the study “Nicotinamide promotes cardiomyocyte derivation and survival through kinase inhibition in human pluripotent stem cells". It has been accepted for publication in Cell Death & Disease.

During the review process, Dr. Chon Lok LEI helped analysis electrophysiology data, so we added him as a co-author. Meanwhile, Dr. Chengcheng Song conducted significant amount wetlab experiments to address reviewers' concerns, so we adjusted her position in the author list. The author lists are below.

The author list in original manuscript: Ya Meng, Zhili Ren, Chengcheng Song, Xiaohong Li, Xiangyu Yang, Nana Ai, Yang Yang, Dongjin Wang, Meixiao Zhan, Jiaxian Wang, Weiwei Liu, Wei Ge, Ligong Lu*, and Guokai Chen*.
The author list in current manuscript: Ya Meng, Chengcheng Song, Zhili Ren, Xiaohong Li, Xiangyu Yang, Nana Ai, Yang Yang, Dongjin Wang, Meixiao Zhan, Jiaxian Wang, Chon Lok LEI, Weiwei Liu, Wei Ge, ligong lu*, and Guokai Chen*.
Please reply this email to confirm whether you are OK with the changes. Thanks for your cooperation.

Best regards,

Guokai Chen & Ya Meng

Re: author change

weiweiliu <weiweiliu@um.edu.mo>

收件人: "孟雅" <mengya_85@163.com>

抄 送: guokaichen <guokaichen@um.edu.mo>

时 间: 2021-10-26 16:18:35

附 件:

Dear Ya,

I confirm that I am fine with the current author list.

Best regards,

Weiwei

Weiwei Liu
Senior Instructor
Operation Manager, Bioimaging and Stem Cell Core Facility
Faculty of Health Sciences, University of Macau
Room 3025, Building E12, Avenida de Universidade, Taipa, Macau SAR
Tel: +853-8822-4982 / 8822-2921

From: 孟雅 <mengya_85@163.com>
Sent: Tuesday, 26 October, 2021 15:43:57
To: weiweiliu
Cc: guokaichen
Subject: author change

Dear Prof. Liu,

Thank you for your contribution to the study “Nicotinamide promotes cardiomyocyte derivation and survival through kinase inhibition in human pluripotent stem cells". It has been accepted for publication in Cell Death & Disease.

During the review process, Dr. Chon Lok LEI helped analysis electrophysiology data, so we added him as a co-author. Meanwhile, Dr. Chengcheng Song conducted significant amount wetlab experiments to address reviewers' concerns, so we adjusted her position in the author list. The author lists are below.

The author list in original manuscript: Ya Meng, Zhili Ren, Chengcheng Song, Xiaohong Li, Xiangyu Yang, Nana Ai, Yang Yang, Dongjin Wang, Meixiao Zhan, Jiaxian Wang, Weiwei Liu, Wei Ge, Ligong Lu*, and Guokai Chen*.
The author list in current manuscript: Ya Meng, Chengcheng Song, Zhili Ren, Xiaohong Li, Xiangyu Yang, Nana Ai, Yang Yang, Dongjin Wang, Meixiao Zhan, Jiaxian Wang, Chon Lok LEI, Weiwei Liu, Wei Ge, ligong lu*, and Guokai Chen*.
Please reply this email to confirm whether you are OK with the changes. Thanks for your cooperation.

Best regards,

Guokai Chen & Ya Meng

Re: author change

weige <weige@um.edu.mo>

收件人: "孟雅" <mengya_85@163.com>

抄　送: guokaichen <guokaichen@um.edu.mo>

时　间: 2021-10-26 16:56:38

附　件:

Yes, the change is fine with me. Wei

Sent from my iPhone

On 26 Oct 2021, at 3:52 PM, 孟雅 <mengya_85@163.com> wrote:

Dear Prof. Ge,

Thank you for your contribution to the study "Nicotinamide promotes cardiomyocyte derivation and survival through kinase inhibition in human pluripotent stem cells". It has been accepted for publication in Cell Death & Disease.

During the review process, Dr. Chon Lok LEI helped analysis electrophysiology data, so we added him as a co-author. Meanwhile, Dr. Chengcheng Song conducted significant amount wetlab experiments to address reviewers' concerns, so we adjusted her position in the author list. The author lists are below.

The author list in original manuscript: Ya Meng, Zhili Ren, Chengcheng Song, Xiaohong Li, Xiangyu Yang, Nana Ai, Yang Yang, Dongjin Wang, Meixiao Zhan, Jiaxian Wang, Weiwei Liu, Wei Ge, Ligong Lu*, and Guokai Chen*.

The author list in current manuscript: Ya Meng, Chengcheng Song, Zhili Ren, Xiaohong Li, Xiangyu Yang, Nana Ai, Yang Yang, Dongjin Wang, Meixiao Zhan, Jiaxian Wang, Chon Lok LEI, Weiwei Liu, Wei Ge, ligong lu*, and Guokai Chen*.

Please reply this email to confirm whether you are OK with the changes. Thanks for your cooperation.

Best regards,

Guokai Chen & Ya Meng

Re:author change

"Ligong Lu" <luligong1969@126.com>

收件人: "孟雅" <mengya_85@163.com>

抄　送: guokaichen@umac.mo

时　间: 2021-10-26 21:50:00

附　件:

I am OK with the changes

--

Ligong Lu, Ph.D, M.D
Zhuhai Interventional Medical Center
Zhuhai Precision Medical Center
Zhuhai People's Hospital, Jinan University
No.79th Kangning Road, Zhuhai, Guangdong 519000,
P. R. China.
luligong1969@126.com

At 2021-10-26 15:53:36, "孟雅" <mengya_85@163.com> wrote:

> Dear Prof. Lu,
>
> Thank you for your contribution to the study “Nicotinamide promotes cardiomyocyte derivation and survival through kinase inhibition in human pluripotent stem cells". It has been accepted for publication in Cell Death & Disease.
>
> During the review process, Dr. Chon Lok LEI helped analysis electrophysiology data, so we added him as a co-author. Meanwhile, Dr. Chengcheng Song conducted significant amount wetlab experiments to address reviewers' concerns, so we adjusted her position in the author list. The author lists are below.
>
> The author list in original manuscript: Ya Meng, Zhili Ren, Chengcheng Song, Xiaohong Li, Xiangyu Yang, Nana Ai, Yang Yang, Dongjin Wang, Meixiao Zhan, Jiaxian Wang, Weiwei Liu, Wei Ge, Ligong Lu*, and Guokai Chen*.
>
> The author list in current manuscript: Ya Meng, Chengcheng Song, Zhili Ren, Xiaohong Li, Xiangyu Yang, Nana Ai, Yang Yang, Dongjin Wang, Meixiao Zhan, Jiaxian Wang, Chon Lok LEI, Weiwei Liu, Wei Ge, ligong lu*, and Guokai Chen*.
>
> Please reply this email to confirm whether you are OK with the changes. Thanks for your cooperation.
>
> Best regards,
>
> Guokai Chen & Ya Meng
